# Serum metabolites and risk of sudden sensorineural hearing loss: A Mendelian randomization study

**DOI:** 10.1016/j.bjorl.2025.101596

**Published:** 2025-04-26

**Authors:** Wenhui Yuan, Yong Liu, Chao Liu, Yuanzheng Qiu

**Affiliations:** a Central South University, Xiangya Hospital, Department of Otolaryngology Head and Neck Surgery, Changsha, China; b Otolaryngology Major Disease Research Key Laboratory of Hunan Province, Changsha, China; c Clinical Research Center for Pharyngolaryngeal Diseases and Voice Disorders in Hunan Province, Changsha, China; d National Clinical Research Center for Geriatric Disorders, Xiangya Hospital, Changsha, China

**Keywords:** Mendelian randomization, Sudden sensorineural hearing loss, Blood metabolites

## Abstract

**Objective:**

Observational studies found that Sudden Sensorineural Hearing Loss (SSNHL) is associated with metabolic disorders, but the causal relationship remains unclear. Here we performed a two-sample Mendelian Randomization (MR) analysis to systematically assess the causation between blood metabolites and SSNHL.

**Methods:**

Summary statistics for blood metabolites were extracted from GWAS data of 7824 European participants on metabolite levels. GWAS data for SSNHL were collected from the FinnGen Consortium R10 release data, which consisted of 3128 cases and 362,353 controls in European populations. The inverse variance weighted method was the primary method for causality analysis while MR-Egger, weighted median and MR-RAPS served as complementary approaches. Cochran’s*Q* test, MR-Egger intercept test, MR-PRESSO, Radial MR, leave-one-out and Steiger test were used for sensitivity analyses. Additionally, we performed metabolic pathway analysis to further explore the potential pathogenesis of SSNHL.

**Results:**

We found that genetically predicted cholesterol, citrate, myristoleate (14:1n5) and tryptophan betaine may increase the risk of SSNHL, while stearate (18:0), pantothenate and glycerol 2-phosphate may act as protective factors for SSNHL. Nevertheless, these metabolites did not reach statistical significance after Bonferroni correction. Sensitivity analyses revealed no evidence of heterogeneity or horizontal pleiotropy. Metabolic pathway analysis revealed the pantothenate and CoA biosynthesis pathway and the citrate cycle pathway potentially related to the pathogenesis of SSNHL.

**Conclusion:**

The findings of our study offer new insights into the role of blood metabolites in the development and pathogenesis of SSNHL and provide potential inspiration for further advancements in clinical settings.

**Level of evidence:**

Level 3.

## Highlights

• Cholesterol, citrate, myristoleate (14:1n5) and tryptophan betaine may cause SSNHL.
• Stearate (18:0), pantothenate and glycerol 2-phosphate may be protective for SSNHL.
• Bonferroni correction had the ability to eliminate these above correlations.
• The above findings can be instructive for future research on SSNHL.

## Introduction

Sudden Sensorineural Hearing Loss (SSNHL), also known as sudden idiopathic hearing loss, is an urgent condition characterized by a rapid onset of hearing loss within 72 h and a minimum decline of 30 dB in the standard Pure-Tone Audiogram (PTA) across three consecutive frequencies.^1^ Recently, SSNHL has garnered attention due to its rising prevalence and persistent detrimental effects on patients’ quality of life.^2^ This disorder typically affects one ear and is frequently accompanied by symptoms such as tinnitus, nausea, and vertigo. Although the precise etiology of SSNHL is not fully understood, currently recognized mechanisms encompass viral infections,^3^ microcirculatory disorders,^4^ autoimmune or inflammatory states.^5^ Therefore, it is imperative to perform thorough examination to identify possible causes and early intervention treatment measures to improving the auditory function for patients with SSNHL.

In recent years, metabolomics has emerged as a valuable tool for elucidating the underlying mechanisms of diseases. Specifically, it can provide insights into disease pathogenesis by identifying modified metabolites or metabolic pathways.^6^, ^7^ There are more and more metabolic biomarkers have been discovered as potential risk factors for SSNHL.^8^ A multicenter case-control study revealed that serum lipid levels are significantly associated with the incidence and prognosis of SSNHL. The identification of dyslipidemia may enhance early assessment and management of SSNHL risks.^9^ Another meta-analysis found that SSNHL patients have a significantly higher level in total cholesterol but not High-Density Lipoprotein Cholesterol (HDL-C) and triglycerides in compare to the controls.^10^ However, these results may be influences by potential confounding factors and the possibility of reverse causality in traditional observational studies, thus leaving the causal relationship between them as an unresolved inquiry. To address these concerns, we have performed our research design by utilizing Mendelian Randomization (MR) analysis. MR is an approach that assessed the causal effects of genetically predicted exposure on outcomes by selecting exposure-associated genetic variants as Instrumental Variables (IVs), with its statistical efficacy ranking second only to Randomized Controlled Trial (RCT). And MR can also effectively reduce the bias caused by confounding factors in causal analysis, because alleles are randomly assigned during pregnancy according to Mendel's second Law, which is similar to a natural RCT.

Therefore, we used MR analysis to determine the causal relationship between human blood metabolites and SSNHL by using publicly available, largescale Genome-Wide Association Studies (GWAS) data. Furthermore, we performed several sensitivity analyses and Steiger test to ascertain the robustness of our results. Finally, metabolic pathway enrichment analysis was conducted via the online tool, MetaboAnalyst 5.0 to explore potential metabolic pathway which would probably contribute to the pathogenesis of SSNHL.

## Methods

### Study design

**Fig. 1:**
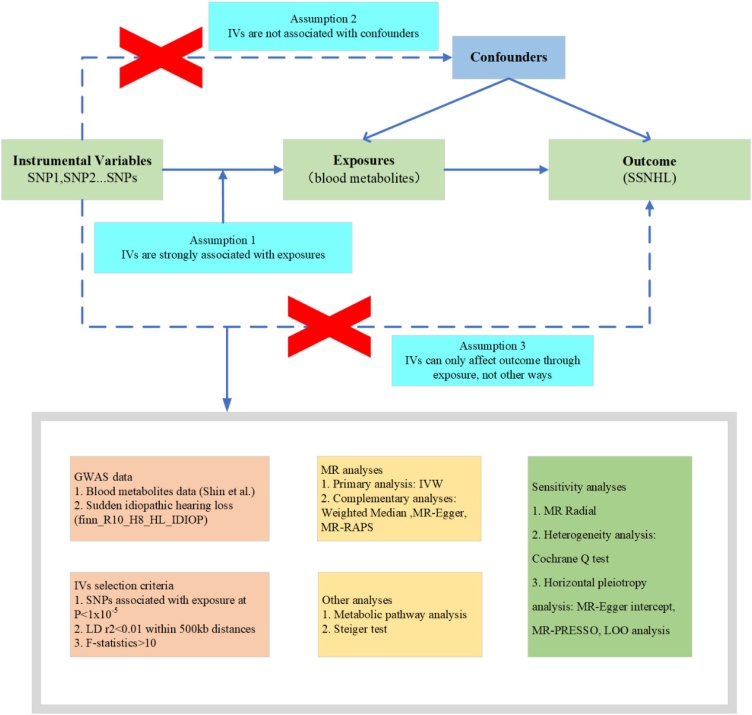
Schematic diagram of this MR study design. MR, Mendelian Randomization; SNP, Single Nucleotide Polymorphisms; SSNHL, Sudden Sensorineural Hearing Loss; IVs, Instrumental Variables, LD, Linkage Disequilibrium; IVW, Inverse Variance Weighted; LOO, Leave-One-Out; MR-RAPS, Mendelian Randomization Robust Adjusted Profile Score.

Fig. 1 presents an overview of the study design and data sources, where we systematically assessed the causal relationship between 486 human serum metabolites and the risk of SSNHL by using a convincing two-sample MR design. Based on the STROBE-MR checklist (Table S1 in Supplementary material),^11^ a rigorous MR study should comply with three major assumptions: 1) The IVs are supposed to have a robust correlation with exposure (i.e., human blood metabolites in this study); 2) The IVs should be unrelated to the outcome (i.e., SSNHL in this study) and free from any known or unknown confounding factors; 3) The IVs’ effects on outcomes are only influenced by the exposures. The presence of horizontal pleiotropy is observed when the IVs exert their impact on the outcome through additional factors.^12^ In this work, we performed MR analyses by using GWAS data of human blood metabolites as exposures and SSNHL as outcome in European population.

### Data sources

GWAS data for human blood metabolites were extracted from the metabolomics GWAS server (http://metabolomics.helmholtz-muenchen.de/gwas/). This dataset was conducted by Shin et al. in 2014, which represents the most comprehensive analysis of genetic effects on human blood metabolites to date.^13^ Specifically, a total of 7824 participants from two European cohorts (KORA F4 cohort and UK Twin cohort) were included in this GWAS analysis and a total of 529 metabolites were analyzed in this study. After strict quality control, 486 metabolites were available for genetic analysis, which consisted of 177 unknown metabolites and 309 known metabolites. Meanwhile, according to the Kyoto Encyclopedia of Genes and Genomes (KEGG) database,^14^ the 309 known metabolites were categorized into eight broad metabolic groups: amino acid, carbohydrates, cofactors and vitamins, energy, lipid, nucleotide, peptide, and xenobiotics. The detailed names of these 486 metabolites are presented in Table S2 (Supplementary material).

The summary data for SSNHL were collected from the FinnGen Consortium R10 release data (https://storage.googleapis.com/finngen-public-data-r10/summary_stats/finngen_R10_H8_HL_IDIOP.gz). This GWAS consisted of 3128 cases and 362,353 controls in European population.

### Selection of instrumental variables (IVs)

MR utilizes genetic variants as IVs from GWAS data to infer the causal relationship between exposure and outcome. In this study, blood metabolites and SSNHL were considered as exposure and outcome, respectively. Firstly, we relaxed the association threshold with *p* < 1.0 × 10^−5^ to select Single Nucleotide Polymorphisms (SNPs) related to blood metabolites, which have been widely used in previous MR studies.^15^ Then, we clumped SNPs by removing linkage disequilibrium (LD, R^2^ < 0.01, and within 500 kb). Secondly, we calculated the F-statistic for each IV and then removed those F-statistic that were below 10, as SNP with F < 10 was deemed weak instruments.^16^ Meanwhile, those SNPs associated with SSNHL and possessing a *p-*value less than 1.0 × 10^−5^ were excluded to adhere to assumption 3. Finally, to maintain the stability of results, we only select the metabolites with more than three or more instrumental SNPs in the following MR analysis.^17^

### MR analysis and sensitivity analysis

The main MR analysis was the Inverse Variance Weighted (IVW) method with the fixed-effects model when there was no significant heterogeneity, otherwise, the random-effects IVW was selected. The IVW assumes no horizontal pleiotropy for all SNPs, making it the most accurate assessment of causation.^18^ To enhance the robustness of the IVW results, we applied three additional methods (MR-Egger, weighted median and MR-RAPS) to further evaluate metabolites with p_ivw_-value less than 0.05, providing more robust estimates under relaxed conditions. The weighted median method estimates the causal effect by calculating the median of the weighted empirical distribution of each SNP effect estimate, even when up to 50% of SNPs were disobeying MR basic assumption.^18^ The MR-Egger approach can detect violations of IVs assumption and provide unaffected effects estimates. The MR-Egger regression can yield unbiased estimates when it aligns with the InSIDE assumption, which states that the strength of IVs is independent of direct effects on outcome.^19^ The MR Robust Adjusted Profile Score (MR-RAPS) method can yield reliable and accurate inferences even in the presence of weak IVs.

Then we conducted five methods to detect and correct for heterogeneity and pleiotropy analysis: Cochran’s *Q* test, MR-Egger intercept, Radial MR, MR-PRESSO, and Leave-One-Out (LOO) analysis. Among these, Cochran’s *Q* test was used for the examination of heterogeneity, and MR-Egger intercept was applied to evaluate the potential bias induced by pleiotropy. Subsequently, Radial MR was applied to identify the outliers, and MR analysis was repeated after removing the biased SNPs, and then MR-PRESSO were utilized to check for the presence of heterogeneous SNPs again. Finally, to ensure the robustness of the aforementioned findings, we performed a LOO analysis to evaluate if a single SNP significantly influenced the MR results by excluding each SNP in turn and performing MR analysis.^19^

Briefly, we rigorously screened blood metabolites for potential causal effects on SSNHL based on the following criteria: 1) Significant *p-*value in the primary analysis (IVW derived *p* < 0.05), 2) Consistent direction and magnitude across four MR approaches (IVW, weighted median, MR-Egger, MR-RAPS), 3) Without heterogeneity or horizontal pleiotropy in MR results, and 4) Minimal influences of MR estimates by a single SNP.

### Metabolic pathway analysis and direction validation

To further investigate the putative role of candidate metabolites, we performed metabolic pathway enrichment analysis by using the MetaboAnalyst 5.0 (https://www.metaboanalyst.ca/), which is an intuitive online tool for efficient metabolomics data analysis. Meanwhile, the Small Molecular Pathway Database (SMPDB) and the KEGG database were used in this study.^20^ Additionally, we conducted the Steiger test to determine if there is reverse causality between blood metabolites and SSNHL. If the explained variance of IVs in SSNHL is stronger than that of blood metabolites, the direction of causal inference may be false.^21^

### Statistical analysis

All MR analyses in this study were performed in R software (Version 4.3.0) by using the TwoSampleMR package, MRPRESSO package, RadialMR package and mr.raps package; *p-*value below 0.05 within IVW method was considered candidate causality between blood metabolites and SSNHL. Additionally, we used Bonferroni correction to re-analyze the MR results by adopting a multiple-test-adjusted threshold of *p* < 1.03 × 10^−4^ (0.05/486). We also reported those metabolites whose *p-*values were between 1.03 × 10^−4^ and 0.05, and we called these suggestive risk factors for SSNHL.

## Results

### MR analysis and sensitivity analysis

After a meticulous selection process for IVs, the filtered IVs contained at least three SNPs, with each SNP having an F-statistic greater than 10 (Table S3 in Supplementary material). In primary analysis, we identified 15 metabolites that had potentially significant causal effect size for SSNHL, which contained 10 known metabolites and 5 unknown metabolites (Table 1). The outliers detected by Radial MR are presented in detail in Table S4 (Supplementary material). Ultimately, we identified 11 eligible candidate metabolites that met the stringent criteria, including 7 known metabolites and 4 unknown metabolites through conducting complementary and sensitivity analyses after removing the outliers (Table 2). Unfortunately, no metabolites were found to still had a significant effect after Bonferroni correction.

**Table 1:** The primary MR analysis of genetically proxied blood metabolites on the risk of SSNHL.

Metabolites	SNPs	MR method	OR (95% CI)	*p-*value
Cholesterol	47	IVW	3.37 (1.33–8.53)	0.0102
Stearate (18:0)	43	IVW	0.41 (0.17‒0.97)	0.0424
Pantothenate	26	IVW	0.43 (0.22‒0.83)	0.0114
Citrate	46	IVW	2.96 (1.28–6.80)	0.0108
X-04499--3,4-dihydroxybutyrate	20	IVW	3.12 (1.21–8.03)	0.0183
X-06351	6	IVW	3.53 (1.17–10.66)	0.0256
Glycerol 2-phosphate	32	IVW	0.42 (0.24‒0.75)	0.0031
Myristoleate (14:1n5)	15	IVW	1.81 (1.05–3.13)	0.0327
Dehydroisoandrosterone Sulfate (DHEA-S)	22	IVW	0.63 (0.42‒0.97)	0.0336
X-11437	15	IVW	0.82 (0.68‒0.98)	0.0322
X-11787	31	IVW	0.35 (0.14‒0.90)	0.0295
X-12189	29	IVW	1.13 (1.03–1.23)	0.0109
Stearidonate (18:4n3)	11	IVW	0.52 (0.29‒0.91)	0.0232
X-12645	19	IVW	0.60 (0.37‒0.97)	0.0366
Tryptophan betaine	13	IVW	1.31 (1.03–1.67)	0.0293

MR, Mendelian Randomization; SSNHL, Sudden Sensorineural Hearing Loss; SNPs, Single Nucleotide Polymorphisms; IVW, Inverse Variance Weighted; OR, Odds Ratio, CI, Confidence Interval.

**Table 2:** The four MR methods results of candidate metabolites on the risk of SSNHL before and after correction.

Metabolites	SNPs	MR method	OR (95% CI)	*p-*value
Amino acid				
X-04499--3,4-dihydroxybutyrate	20	IVW	3.12 (1.21–8.03)	0.0183
		WM	4.06 (1.09–15.14)	0.0371
		MR-Egger	0.79 (0.01–42.58)	0.9107
		MR-RAPS	3.22 (1.18–8.78)	0.0220
Tryptophan betaine	13	IVW	1.31 (1.03–1.67)	0.0293
		WM	1.35 (0.98–1.86)	0.0631
		MR-Egger	1.23 (0.59–2.55)	0.5969
		MR-RAPS	1.31 (1.01–1.70)	0.0383
Cofactors and vitamins				
Pantothenate	26	IVW	0.43 (0.22‒0.83)	0.0114
		WM	0.49 (0.18–1.38)	0.1795
		MR-Egger	0.34 (0.10–1.13)	0.0915
		MR-RAPS	0.42 (0.21‒0.83)	0.0128
Energy				
Citrate	46	IVW	2.96 (1.28–6.80)	0.0108
Before correction		WM	3.02 (0.89–10.23)	0.0762
		MR-Egger	2.87 (0.28–29.76)	0.3807
		MR-RAPS	3.08 (1.29–8.60)	0.0116
Citrate	43	IVW	7.47 (0.63–88.24)	0.0049
After correction		WM	3.15 (0.94–10.58)	0.0637
		ME	7.47 (0.63–88.24)	0.1179
		MRRAPS	3.58 (1.43–8.97)	0.0064
Lipid				
Cholesterol	47	IVW	3.37 (1.33–8.53)	0.0102
Before correction		WM	3.82 (1.00–14.54)	0.0492
		MR-Egger	6.19 (0.51–75.62)	0.1602
		MR-RAPS	3.53 (1.33–9.36)	0.0114
Cholesterol	46	IVW	2.84 (1.11–7.24)	0.0287
After correction		WM	3.81 (0.94–15.44)	0.0611
		MR-Egger	9.13 (0.74–113.11)	0.0920
		MR-RAPS	1.09 (2.93–7.86)	0.0325
Stearate (18:0)	43	IVW	0.41 (0.17‒0.97)	0.0424
Before correction		WM	0.34 (0.10–1.12)	0.0760
		MR-Egger	0.44 (0.04–4.75)	0.5053
		MR-RAPS	0.40 (0.16‒0.99)	0.0471
Stearate (18:0)	41	IVW	0.41 (0.17‒0.98)	0.0446
After correction		WM	0.32 (0.09–1.11)	0.0737
		MR-Egger	0.38 (0.03–4.10)	0.4270
		MR-RAPS	0.40 (0.16–1.01)	0.0532
Dehydroisoandrosterone Sulfate (DHEA-S)	22	IVW	0.63 (0.42‒0.97)	0.0336
Before correction		WM	0.69 (0.37–1.29)	0.2443
		MR-Egger	0.90 (0.30–2.73)	0.8596
		MR-RAPS	0.62 (0.40‒0.95)	0.0297
Dehydroisoandrosterone Sulfate (DHEA-S)	21	IVW	0.72 (0.47–1.10)	0.1261
After correction		WM	0.70 (0.38–1.29)	0.2502
		MR-Egger	0.85 (0.29–2.48)	0.7662
		MR-RAPS	0.71 (0.46–1.11)	0.1308
Myristoleate (14:1n5)	15	IVW	1.81 (1.05–3.13)	0.0327
		WM	1.70 (0.72–4.00)	0.2258
		MR-Egger	1.98 (0.67–5.87)	0.2413
		MR-RAPS	1.84 (1.03–3.33)	0.0403
Stearidonate (18:4n3)	11	IVW	0.52 (0.29‒0.91)	0.0232
		WM	0.76 (0.35–1.63)	0.4775
		MR-Egger	1.30 (0.38–4.52)	0.6847
		MR-RAPS	0.50 (0.27‒0.93)	0.0291
Xenobiotics				
Glycerol 2-phosphate	32	IVW	0.42 (0.24‒0.75)	0.0031
Before correction		WM	0.39 (0.16‒0.94)	0.0366
		MR-Egger	0.50 (0.18–1.42)	0.2056
		MR-RAPS	0.40 (0.23‒0.71)	0.0018
Glycerol 2-phosphate	30	IVW	0.38 (0.22‒0.66)	0.0006
After correction		WM	0.38 (0.15‒0.96)	0.0407
		MR-Egger	0.36 (0.14‒0.95)	0.0492
		MR-RAPS	0.37 (0.20‒0.67)	0.0011
Unknown				
X-06351	6	IVW	3.53 (1.17–10.66)	0.0256
		WM	3.56 (0.93–13.70)	0.0645
		MR-Egger	3.13 (0.38–25.61)	0.3466
		MR-RAPS	3.53 (1.06–11.83)	0.0405
X-11437	15	IVW	0.82 (0.68‒0.98)	0.0322
		WM	0.84 (0.66–1.08)	0.1735
		MR-Egger	0.89 (0.68–1.16)	0.3944
		MR-RAPS	0.81 (0.67‒0.98)	0.0300
X-11787	31	IVW	0.35 (0.14‒0.90)	0.0295
		WM	0.73 (0.19–2.74)	0.6400
		MR-Egger	0.50 (0.09–2.80)	0.4368
		MR-RAPS	0.35 (0.13‒0.91)	0.0315
X-12189	29	IVW	1.13 (1.03–1.23)	0.0109
Before correction		WM	1.13 (0.99–1.28)	0.0600
		MR-Egger	1.06 (0.93–1.22)	0.3756
		MR-RAPS	1.13 (1.03–1.24)	0.0124
X-12189	27	IVW	1.14 (1.04–1.26)	0.0044
After correction		WM	1.14 (1.00–1.30)	0.0481
		MR-Egger	1.10 (0.96–1.26)	0.1994
		MR-RAPS	1.15 (1.04–1.27)	0.0067
X-12645	19	IVW	0.60 (0.37‒0.97)	0.0366
		WM	0.56 (0.28–1.13)	0.1034
		MR-Egger	1.07 (0.33–3.53)	0.9080
		MR-RAPS	0.59 (0.35‒0.99)	0.0450

MR, Mendelian Randomization; SSNHL, Sudden Sensorineural Hearing Loss; SNPs, Single Nucleotide Polymorphisms; IVW, Inverse Variance Weighted; WM, Weighted Median; MR-RAPS, Mendelian Randomization Robust Adjusted Profile Score; OR, Odds Ratio, CI, Confidence Interval.

**Fig. 2:**
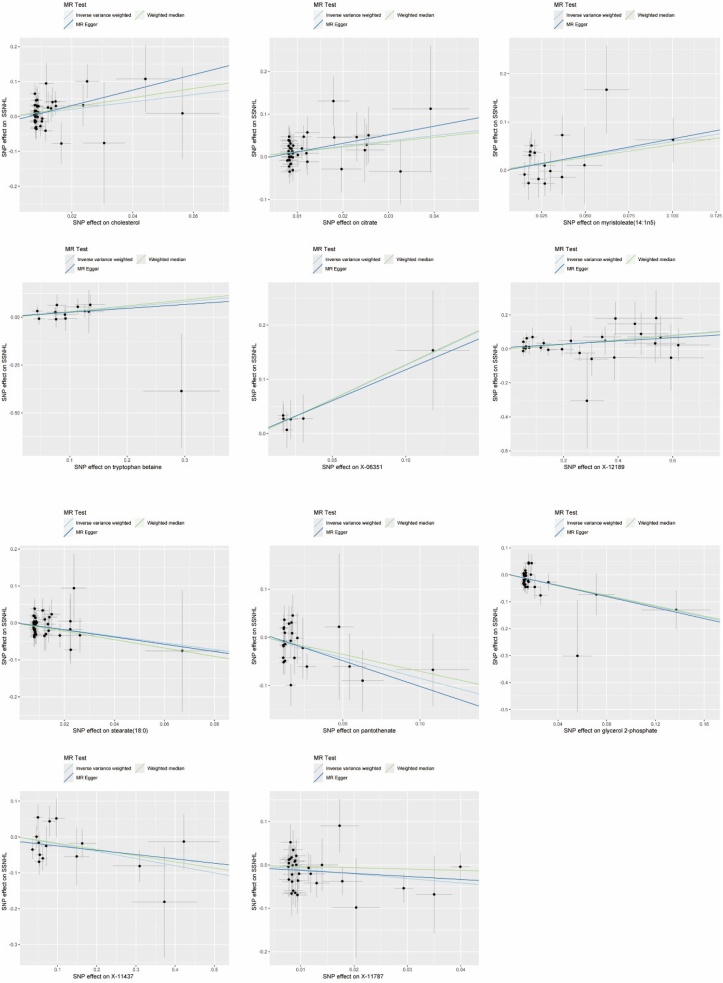
Scatter plot of the causality of 11 candidate metabolites on SSNHL.

Of the eleven candidate metabolites, six may be associated with an increased risk of SSNHL: cholesterol (OR = 2.84, 95% CI: 1.11–7.24, *p* = 0.0287), citrate (OR = 7.47, 95% CI: 0.63–88.24, *p* = 0.0049), myristoleate (14:1n5) (OR = 1.81, 95% CI: 1.05–3.13, *p* = 0.0327), tryptophan betaine (OR = 1.31, 95% CI: 1.03–1.67, *p* = 0.0293), X-06351 (OR = 3.53, 95% CI: 1.17–10.66, *p* = 0.0256), X-12189 (OR = 1.14, 95% CI: 1.04–1.26, *p* = 0.0044); the remaining five metabolites have a suggestive protective role on the pathogenesis of SSNHL: stearate (18:0) (OR = 0.41, 95% CI: 0.17‒0.98, *p* = 0.0446), pantothenate (OR = 0.43, 95% CI: 0.22‒0.83, *p* = 0.0114), glycerol 2-phosphate (OR = 0.42, 95% CI: 0.24‒0.75, *p* = 0.0031), X-11437 (OR = 0.82, 95% CI: 0.68‒0.98, *p* = 0.0322), X-11787 (OR = 0.35, 95% CI: 0.14‒0.90, *p* = 0.0295) (Fig. 2 and Fig. S1-S2).

The presence of statistical heterogeneity was not detected by Cochran’s *Q* test (Table 3). Meanwhile, both MR-Egger intercepts (Table 3) and the MR-PRESSO global tests (Table S5 in Supplementary material) consistently demonstrated no horizontal pleiotropy. Furthermore, LOO analysis found no high-influence SNPs that would have biased the pooled effect estimates (Fig. S3-S4).

**Table 3:** The results of Cochran’s *Q* test and MR-Egger intercept test.

Metabolites	Category	Heterogeneity		Pleiotropy	
		*Q*	*p-*value	Intercept	*p-*value
X-04499--3,4-dihydroxybutyrate	Amino Acid	14.6917	0.7420	0.0209	0.4968
Tryptophan betaine	Amino Acid	5.6555	0.9324	0.0059	0.8535
Pantothenate	Cofactors and vitamins	25.6529	0.4263	0.0050	0.6674
Citrate	Energy	27.5676	0.9580	−0.0086	0.5187
Cholesterol	Lipid	31.6597	0.9336	−0.0129	0.3328
Stearate (18:0)	Lipid	21.7863	0.9916	0.0008	0.9474
Dehydroisoandrosterone Sulfate (DHEA-S)	Lipid	14.6128	0.7981	−0.0057	0.7456
Myristoleate (14:1n5)	Lipid	13.9463	0.4537	−0.0031	0.8588
Stearidonate (18:4n3)	Lipid	9.6567	0.4711	−0.0329	0.1346
Glycerol 2-phosphate	Xenobiotics	23.8807	0.7348	0.0010	0.9124
X-12189	Unknown	16.5308	0.9226	0.0094	0.4584
X-06351	Unknown	0.6111	0.6111	0.0034	0.9033
X-11437	Unknown	16.0890	0.3080	−0.0128	0.4344
X-11787	Unknown	22.8568	0.8211	−0.0057	0.6460
X-12645	Unknown	10.9295	0.8973	−0.0183	0.3023

Q, Q statistic.

### Metabolic pathway analysis and Steiger test

In this study, we selected 7 known candidate metabolites that may be implicated in the mechanism of SSNHL pathogenesis for further metabolic pathway enrichment analysis, and the significance threshold for the final pathway analysis was set at 0.05.

Our results identified eight metabolic pathways that may potentially contribute to the development of SSNHL, with two of them exhibiting statistically significant disparities. These pathways include the pantothenate and CoA biosynthesis pathway (*p* < 0.005) and the citrate cycle (TCA cycle) pathway (*p* < 0.05) (Table 4).

**Table 4:** Metabolic pathways enrichment analysis of seven known candidate metabolites.

Metabolic pathways	Involved metabolites	*p-*value
Pantothenate and CoA biosynthesis	Pantothenate	0.0499
Citrate cycle (TCA cycle)	Citrate	0.0499
Alanine, aspartate and glutamate metabolism	Citrate	0.0693
Glyoxylate and dicarboxylate metabolism	Citrate	0.0789
Biosynthesis of unsaturated fatty acids	Stearate	0.0884
Steroid biosynthesis	Cholesterol	0.1002
Primary bile acid biosynthesis	Cholesterol	0.1119
Steroid hormone biosynthesis	Cholesterol	0.2035

Additionally, the Steiger test confirmed that there were no reverse causal effects violating the causality between genetically predicted blood metabolites and SSNHL (Table S6 in Supplementary material).

## Discussion

This study represents the first attempt to investigate the causal relationship between human blood metabolites and SSNHL via two-sample MR analysis based on large-scale GWAS data from independent consortiums of European populations. Our results revealed that eleven serum metabolites may exhibit distinct causal association with SSNHL, with seven known-chemical identity metabolites. Specifically, cholesterol, citrate, myristoleate (14:1n5) and tryptophan betaine were observed to have suggestive positive causal relationships with the risk of SSNHL. In contrast, serum stearate (18:0), pantothenate and glycerol 2-phosphate levels may act as protective factors for developing SSNHL, as evidenced by OR-value < 1. Meanwhile, we identified two metabolic pathways ‒ the pantothenate and CoA biosynthesis pathway and the TCA cycle pathway ‒ as potentially playing key roles in the pathogenesis of SSNHL. These findings may offer a theoretical foundation for the exploration of biomarkers in SSNHL. Moreover, all statistical methods consistently support the stability and reliability of our findings. Therefore, monitoring and focusing on these serum metabolites should be incorporated into SSNHL prevention and intervention strategies, which will contribute to deepen into the investigation of the pathogenesis and treatment in SSNHL.

Metabolomics is a promising and powerful approach for characterizing metabolic diversity and identifying potential biomarkers for disease phenotype and prognosis prediction. Recently, the role of regulating metabolic networks in inner ear diseases has garnered significant attention, emerging as a novel avenue to elucidate the pathology of the inner ear.^22^, ^23^ Although these studies have provided compelling evidence for the involvement of metabolites in the biological mechanisms of SSNHL, which is beneficial for its treatment, their contribution to early screening and prevention remains limited due to the ambiguous causal relationship between them.^8^ Therefore, we conducted a critical MR study with the hope of clarifying this causal relationship between blood metabolites and SSNHL as well as identifying the metabolic pathways involved, thus providing a reference point for SSNHL screening and intervention.

First and foremost, the lack of high-quality studies necessitates caution in illuminating the causal relationships between candidate metabolites and SSNHL. A recently MR study has found that reduced serum HDL-C levels serve as a risk factor for SSNHL,^24^ and cholesterol we talk about here refers to the total serum cholesterol. In this study, we found that high levels of serum cholesterol may increase the prevalence of SSNHL, which was consistent with previous observational studies.^25^, ^26^ The reason for this may be that elevated serum cholesterol hampers the release of nitric oxide, which in turn impairs the dilation of inner ear blood vessels and consequently affects the microcirculation within the inner ear.^27^

We also confirmed that genetic predisposition to higher levels of citrate, myristoleate (14:1n5) and tryptophan betaine may be detrimental to SSNHL. To date, there are no reports about the correlation between citrate and SSNHL. It is well-known that citrate, as an important intermediate in the TCA cycle, plays a very important role in the energy metabolism of cells. When there is a fluctuation in citrate levels, it can lead to the insufficiency of ATP synthesis and the disturbance of energy metabolism by impacting mitochondrial function and oxidative phosphorylation process. Abnormal citrate metabolism has also been linked to various disorders, including metabolic diseases and neurodegenerative conditions. In terms of deafness, several studies revealed that decreased activity or mutation of citrate synthase, the key enzyme in citric acid synthesis, may contribute to age-related hearing loss by affecting mitochondrial dysfunction.^28^, ^29^ This seems to imply an association between citrate and the risk of SSNHL although further studies are needed to validate this.

Myristoleate (14:1n5), as a saturated fatty acid in the process of fatty synthesis, can promote inflammation and angiogenesis.^30^, ^31^ A prospective case-control study on the etiology of SSNHL found that inflammation plays a very important role in the onset of SSNHL.^32^, ^33^ Furthermore, the previous studies suggested that inner hair cells exposed to pathophysiological stimuli undergo disturbed fatty acid metabolism, which can lead to oxidative stress injury and apoptosis in the inner hair cells, ultimately resulting in acute hearing loss.^23^, ^34^, ^35^ Our MR study found that higher genetic susceptibility to myristoleate (14:1n5) may increase the incidence of SSNHL. It is speculated that myristoleate (14:1n5) may exacerbate the risk of SSNHL through disordering fatty acid metabolism and promoting inflammation mechanism, but the exact biological mechanism requires further exploration.

Currently, there is insufficient research on the metabolism and physiological function of tryptophan betaine. Preliminary studies have demonstrated a certain anti-inflammatory effect of tryptophan betaine on endothelial cell injury.^36^ However, the latest MR study has revealed its potential as a risk factor for breast cancer, particularly ER-positive breast cancer.^37^ Our findings suggest a potential negative correlation between tryptophan betaine and the risk of SSNHL, although the specific mechanism remains unclear, it provides clues for further study of their relationship.

This study additionally recognized some metabolites may act as protective factors for SSNHL, namely, stearate (18:0), pantothenate and glycerol 2-phosphate. Stearate is a blood metabolite in the fatty acid metabolism pathway, and as a protective factor of SSNHL, it has been concluded to have a negative causal association with Irritable Bowel Syndrome (IBD) and Gastroesophageal Reflux Disease (GERD).^38^, ^39^ For pantothenate, it is also known as vitamin B5, is a vital precursor for the synthesis of CoA, which has been demonstrated to exert a protective effect against cisplatin-induced ototoxicity.^40^ Our MR study revealed the protective role of pantothenate in SSNHL development, with metabolic pathway analysis indicating that pantothenate and CoA biosynthesis as a potential involved pathway in the development of SSNHL. Notably, glycerol 2-phosphate, a xenobiotic, exhibited a suggestive inverse relationship with SSNHL. This correlation suggests that environmental exposures may significantly impact the metabolic landscape of SSNHL, thereby influencing disease susceptibility. The regulation of metabolic pathways by xenobiotics, which are often associated with external factors like diet or pollutants, reveals an unexplored aspect in the complex nature of SSNHL.

It is worth noting that the association between the above candidate metabolites and SSNHL did not retain statistical significance following Bonferroni correction, a method widely adopted in MR analysis to control for false positives at the cost of reduced statistical power. Nonetheless, the pre-correction significance suggests potential mechanistic links that merit further exploration. Given the high intercorrelations among serum metabolites and the possibility of biologically relevant pathways, these findings underscore the need for additional studies to elucidate the physiological and pathological mechanisms underlying these suggestive relationships.

This MR analysis has several noteworthy advantages. Firstly, this work covered a wide range of blood metabolites as exposure factors for MR analysis, aiming to investigate the metabolic characteristics that lead to SSNHL. In contrast to previous studies, our research has identified a prospective association between serum metabolites and an elevated risk of SSNHL, thereby mitigating the inherent limitations associated with observational studies. Moreover, it provides a better understanding of the inherent association between serum metabolites and SSNHL, which is helpful for effective screening and prevention strategies in clinical settings of SSNHL. However, several limitations that may impact the reliability of the reported results need to be taken into consideration. Firstly, the accuracy of MR estimation is partially dependent on the sample size of cases, and in our study, the sample size of SSNHL was limited. Furthermore, there was a dearth of detailed stratification data such as gender, age and other information like specific diagnostic classification and prognosis for SSNHL. Additionally, the predominantly European composition of the study cohort necessitates cautious extrapolation of these findings to other populations due to potential variations in genetic and environmental factors across different ethnicities, which may result in divergent outcomes regarding the interaction between blood metabolites and SSNHL.

Consequently, the precise mechanisms by which metabolites exert their impact on SSNHL remain insufficiently investigated. Further dedicated research efforts are necessary to elucidate these complex molecular mechanisms.

## Conclusion

In summary, our study identified 11 suggestive causal relationships involving 7 known metabolites and determined the pantothenate and CoA biosynthesis pathway and the TCA cycle pathway potentially related to the pathogenesis of SSNHL. These results suggest that SSNHL is a complex and multifactorial condition that requires timely medical intervention for optimal outcomes, and it also underscore the potential significance of monitoring and placing emphasis on serum metabolites levels as a method of both preventing and treating SSNHL.

## Ethics statement

All data in this study are available in publicly available databases. No additional ethical approval was required.

## Funding

This study was supported by the 10.13039/501100001809National Natural Science Foundation of China (nº 82103631, nº 82073009), the Natural Science Foundation of Hunan Province (nº 2021JJ41005).

## Data availability

We used publicly available databases, and all data mentioned in the manuscript can be found on the website provided in the article.

## Declaration of competing interest

The authors declare no conflicts of interest.

## Footnotes

Appendix A Supplementary material related to this article can be found, in the online version, at doi:https://doi.org/10.1016/j.bjorl.2025.101596.
