# Supplementary material for: Serum metabolites and risk of sudden sensorineural hearing loss: A Mendelian randomization study
Source: Braz J Otorhinolaryngol. 2025 Apr 26;91(4):101596. doi: 10.1016/j.bjorl.2025.101596 (PMC12056394; doi:10.1016/j.bjorl.2025.101596)
Supplement: Supplementary file 1 [file mmc1.docx]

Supplementary materials

Fig. S1. Forest plot for the six serum metabolites that may be risk factors for SSNHL.

Fig. S2. Forest plot for the five serum metabolites that may be protective factors for SSNHL.

Fig. S3. Leave-one-out plots for the causal association between six risk serum metabolites and SSNHL.

Fig. S4. Leave-one-out plots for the causal association between five protective serum metabolites and SSNHL.

Supplemental table 1. STROBE-MR checklist of recommended items to address in reports of Mendelian randomization studies.

Supplemental table 2. Detailed information for each of the 486 blood metabolites.

Supplemental table 3. Summary information on the SNPs used as instrumental variables.

Supplemental table 4. Outliers identified by Radial MR.

Supplemental table 5. The results of MR-PRESSO after removing outliers.

Supplemental table 6. Estimation of the Steiger direction test from 15 blood metabolites to SSNHL.


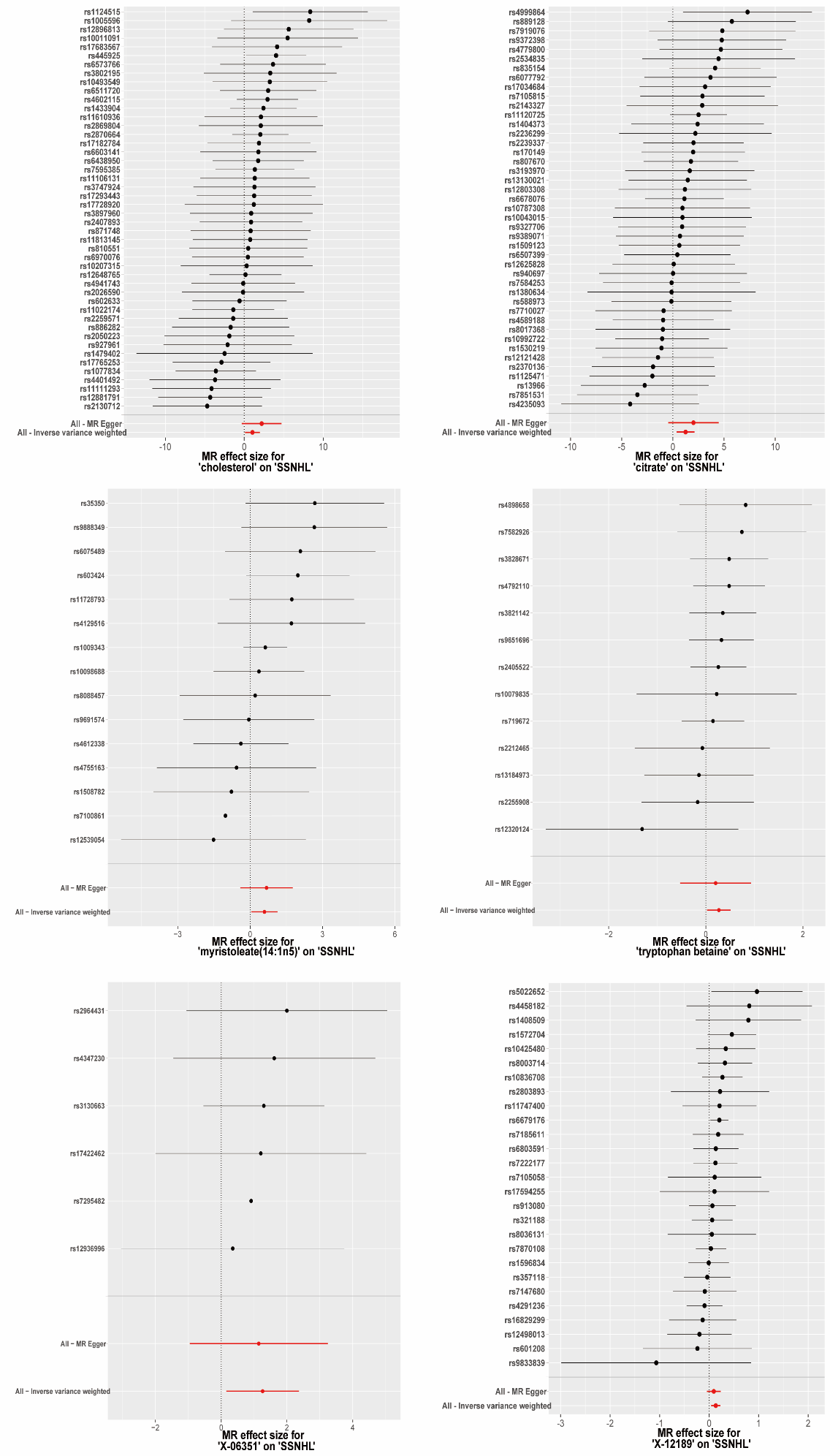


Fig. S1. Forest plot for the six serum metabolites that may be risk factors for SSNHL.


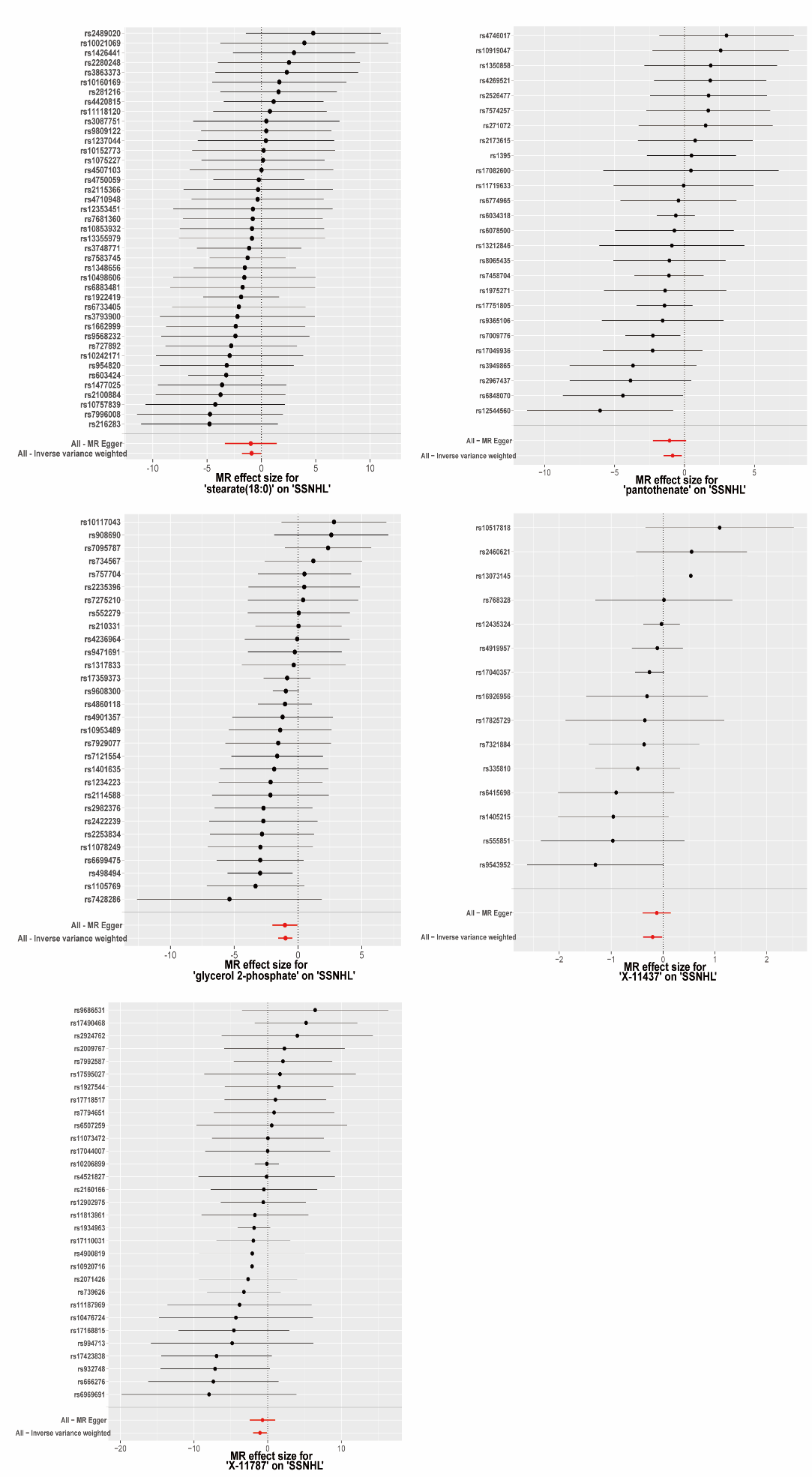


Fig. S2. Forest plot for the five serum metabolites that may be protective factors for SSNHL.


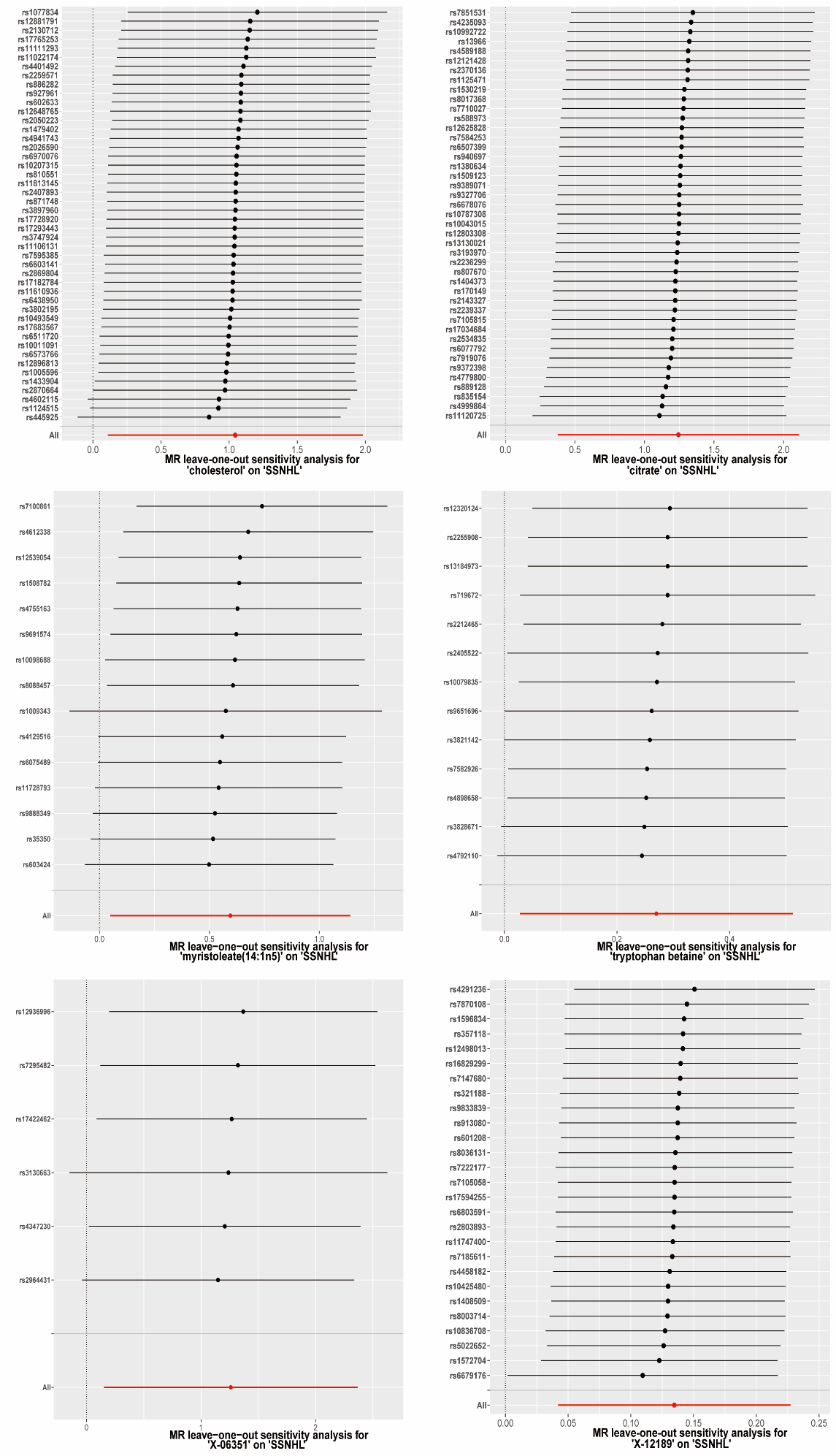


Fig. S3. Leave-one-out plots for the causal association between six risk serum metabolites and SSNHL.


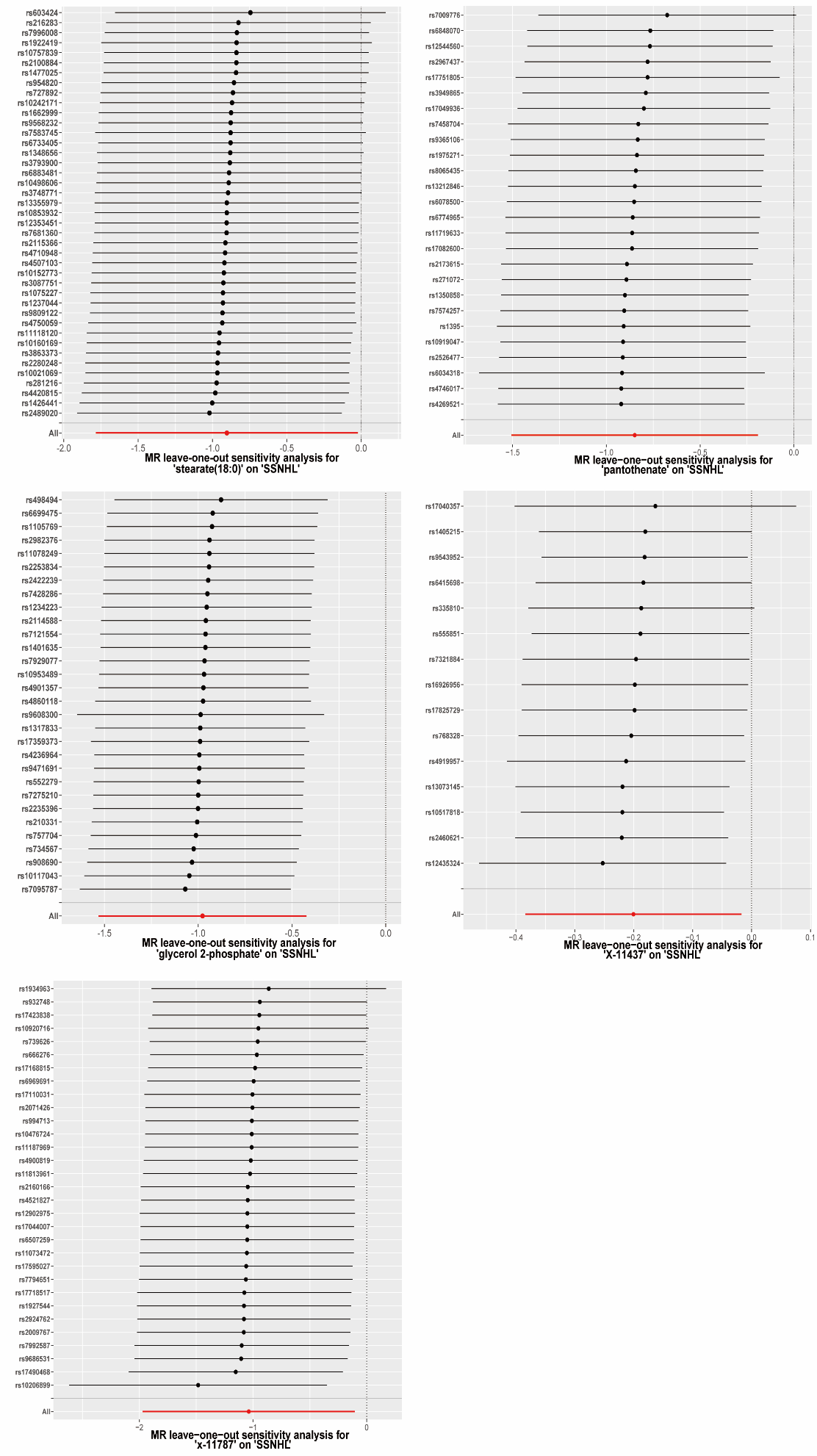


Fig. S4. Leave-one-out plots for the causal association between five protective serum metabolites and SSNHL.

**Supplemental table 1. STROBE-MR checklist of recommended items to address in reports of Mendelian randomization studies**^1^ ^2^

| **Item No.** | **Section** | **Checklist item** | **Page No.** | **Relevant text from manuscript** |
| --- | --- | --- | --- | --- |
| 1 | **TITLE and ABSTRACT** | Indicate Mendelian randomization (MR) as the study’s design in the title and/or the abstract if that is a main purpose of the study | 1,3 | Title, Abstract |
|  | **INTRODUCTION** |  |  |  |
| 2 | **Background** | Explain the scientific background and rationale for the reported study. What is the exposure? Is a potential causal relationship between exposure and outcome plausible? Justify why MR is a helpful method to address the study question | 5 | Introduction |
| 3 | **Objectives** | State specific objectives clearly, including pre-specified causal hypotheses (if any). State that MR is a method that, under specific assumptions, intends to estimate causal effects | 5 | Introduction |
|  | **METHODS** |  |  |  |
| 4 | **Study design and data sources** | Present key elements of the study design early in the article. Consider including a table listing sources of data for all phases of the study. For each data source contributing to the analysis, describe the following: |  |  |
|  | a) | Setting: Describe the study design and the underlying population, if possible. Describe the setting, locations, and relevant dates, including periods of recruitment, exposure, follow-up, and data collection, when available. | 7 | Figure 1 and 2.1 study design paragraph |
|  | b) | Participants: Give the eligibility criteria, and the sources and methods of selection of participants. Report the sample size, and whether any power or sample size calculations were carried out prior to the main analysis | 7 | 2.2 data sources paragraphs |
|  | c) | Describe measurement, quality control and selection of genetic variants | 8 | 2.3 Selection of instrumental variables paragraphs |
|  | d) | For each exposure, outcome, and other relevant variables, describe methods of assessment and diagnostic criteria for diseases | 8 | 2.4 MR analysis and sensitivity analysis paragraphs |
|  | e) | Provide details of ethics committee approval and participant informed consent, if relevant |  | Ethic statements were claimed in the involving GWAS studies. |
| 5 | **Assumptions** | Explicitly state the three core IV assumptions for the main analysis (relevance, independence and exclusion restriction) as well assumptions for any additional or sensitivity analysis | 7 | 2.1 study design paragraph |
| 6 | **Statistical methods: main analysis** | Describe statistical methods and statistics used |  |  |
|  | a) | Describe how quantitative variables were handled in the analyses (i.e., scale, units, model) | 8 | 2.3 Selection of instrumental variables paragraph |
|  | b) | Describe how genetic variants were handled in the analyses and, if applicable, how their weights were selected | 8 | 2.3 Selection of instrumental variables paragraph |
|  | c) | Describe the MR estimator (e.g. two-stage least squares, Wald ratio) and related statistics. Detail the included covariates and, in case of two-sample MR, whether the same covariate set was used for adjustment in the two samples | 8 | 2.4 MR analysis and sensitivity paragraph |
|  | d) | Explain how missing data were addressed | 8 | 2.3 Selection of instrumental variables paragraph |
|  | e) | If applicable, indicate how multiple testing was addressed | 10 | 2.6 statistical analysis paragraph |
| 7 | **Assessment of assumptions** | Describe any methods or prior knowledge used to assess the assumptions or justify their validity | 8 | 2.4 MR analysis and sensitivity analysis and 2.5 Metabolic pathways and direction validation paragraphs |
| 8 | **Sensitivity analyses and additional analyses** | Describe any sensitivity analyses or additional analyses performed (e.g. comparison of effect estimates from different approaches, independent replication, bias analytic techniques, validation of instruments, simulations) | 8 | 2.4 MR analysis and sensitivity analysis paragraph |
| 9 | **Software and pre-registration** |  |  |  |
|  | a) | Name statistical software and package(s), including version and settings used | 10 | 2.6 statistical analysis paragraph |
|  | b) | State whether the study protocol and details were pre-registered (as well as when and where) |  | No statement |
|  | **RESULTS** |  |  |  |
| 10 | **Descriptive data** |  |  |  |
|  | a) | Report the numbers of individuals at each stage of included studies and reasons for exclusion. Consider use of a flow diagram |  | Not applicable, as we used summary statistics from previously published genome-wide association studies. We cite these accordingly. Furthermore, there is no sample overlap between the exposure and outcome studies. |
|  | b) | Report summary statistics for phenotypic exposure(s), outcome(s), and other relevant variables (e.g. means, SDs, proportions) |  | Not applicable, as we used summary statistics from public genome-wide association studies. We cite these accordingly. Furthermore, there is no sample overlap between the exposure and outcome studies. |
|  | c) | If the data sources include meta-analyses of previous studies, provide the assessments of heterogeneity across these studies |  | Not applicable, as we used summary statistics from public genome-wide association studies. We cite these accordingly. Furthermore, there is no sample overlap between the exposure and outcome studies. |
|  | d) | For two-sample MR:  i.  Provide justification of the similarity of the genetic variant-exposure associations between the exposure and outcome samples  ii.  Provide information on the number of individuals who overlap between the exposure and outcome studies |  | Not applicable, as we used summary statistics from public genome-wide association studies. We cite these accordingly. Furthermore, there is no sample overlap between the exposure and outcome studies. |
| 11 | **Main results** |  |  |  |
|  | a) | Report the associations between genetic variant and exposure, and between genetic variant and outcome, preferably on an interpretable scale | 11 | 3.1 MR analysis and sensitivity analysis paragraph |
|  | b) | Report MR estimates of the relationship between exposure and outcome, and the measures of uncertainty from the MR analysis, on an interpretable scale, such as odds ratio or relative risk per SD difference | 11 | 3.1 MR analysis and sensitivity analysis paragraph |
|  | c) | If relevant, consider translating estimates of relative risk into absolute risk for a meaningful time period | 11 | 3.1 MR analysis and sensitivity analysis paragraph |
|  | d) | Consider plots to visualize results (e.g. forest plot, scatterplot of associations between genetic variants and outcome versus between genetic variants and exposure) | 11 | 3.1 MR analysis and sensitivity analysis paragraph |
| 12 | **Assessment of assumptions** |  |  |  |
|  | a) | Report the assessment of the validity of the assumptions | 11 | 3.1 MR analysis and sensitivity analysis paragraph |
|  | b) | Report any additional statistics (e.g., assessments of heterogeneity across genetic variants, such as *I^2^*, Q statistic or E-value) | 11 | 3.1 MR analysis and sensitivity analysis paragraph |
| 13 | **Sensitivity analyses and additional analyses** |  |  |  |
|  | a) | Report any sensitivity analyses to assess the robustness of the main results to violations of the assumptions | 11 | 3.1 MR analysis and sensitivity analysis paragraph |
|  | b) | Report results from other sensitivity analyses or additional analyses | 11 | 3.1 MR analysis and sensitivity analysis paragraph and 3.2 Metabolic pathway analysis and Steiger test paragraph |
|  | c) | Report any assessment of direction of causal relationship (e.g., bidirectional MR) | 11 | 3.23.2 Metabolic pathway analysis and Steiger test paragraph |
|  | d) | When relevant, report and compare with estimates from non-MR analyses |  | Not applicable |
|  | e) | Consider additional plots to visualize results (e.g., leave-one-out analyses) | 11 | 3.1 MR analysis and sensitivity analysis paragraph |
|  | **DISCUSSION** |  |  |  |
| 14 | **Key results** | Summarize key results with reference to study objectives | 13 | Discussion-paragraph 1 |
| 15 | **Limitations** | Discuss limitations of the study, taking into account the validity of the IV assumptions, other sources of potential bias, and imprecision. Discuss both direction and magnitude of any potential bias and any efforts to address them | 16 | Discussion-paragraph 8 |
| 16 | **Interpretation** |  |  |  |
|  | a) | Meaning: Give a cautious overall interpretation of results in the context of their limitations and in comparison, with other studies | 13 | Discussion-paragraph 1 |
|  | b) | Mechanism: Discuss underlying biological mechanisms that could drive a potential causal relationship between the investigated exposure and the outcome, and whether the gene-environment equivalence assumption is reasonable. Use causal language carefully, clarifying that IV estimates may provide causal effects only under certain assumptions | 13-15 | Discussion-paragraph 3-7 |
|  | c) | Clinical relevance: Discuss whether the results have clinical or public policy relevance, and to what extent they inform effect sizes of possible interventions | 15-16 | Discussion-paragraph 8 |
| 17 | **Generalizability** | Discuss the generalizability of the study results (a) to other populations, (b) across other exposure periods/timings, and (c) across other levels of exposure | 15-16 | Discussion-paragraph 8 |
|  | **OTHER INFORMATION** |  |  |  |
| 18 | **Funding** | Describe sources of funding and the role of funders in the present study and, if applicable, sources of funding for the databases and original study or studies on which the present study is based | 1 | Funding |
| 19 | **Data and data sharing** | Provide the data used to perform all analyses or report where and how the data can be accessed, and reference these sources in the article. Provide the statistical code needed to reproduce the results in the article, or report whether the code is publicly accessible and if so, where | 7-8 | GWAS summary statistics for blood metabolites can be accessed at (<http://metabolomics.helmholtz-muenchen.de/gwas/>).  GWAS summary statistics for sudden sensorineural hearing loss can be downloaded from (<https://storage.googleapis.com/finngen-public-data-r10/summary_stats/finngen_R10_H8_HL_IDIOP.gz>) |
| 20 | **Conflicts of Interest** | All authors should declare all potential conflicts of interest | 1 | The authors declare no conflict of interest. |

This checklist is copyrighted by the Equator Network under the Creative Commons Attribution 3.0 Unported (CC BY 3.0) license.

1. Skrivankova VW, Richmond RC, Woolf BAR, Yarmolinsky J, Davies NM, Swanson SA, et al. Strengthening the Reporting of Observational Studies in Epidemiology using Mendelian Randomization (STROBE-MR) Statement. JAMA. 2021;under review.

2. Skrivankova VW, Richmond RC, Woolf BAR, Davies NM, Swanson SA, VanderWeele TJ, et al. Strengthening the Reporting of Observational Studies in Epidemiology using Mendelian Randomisation (STROBE-MR): Explanation and Elaboration. BMJ. 2021;375:n2233.

**Supplemental table 2. Detailed information for each of the 486 blood metabolites.**

| **Metabolite ID** | **Metabolites** | **Status** | **Super-pathway** |
| --- | --- | --- | --- |
| M00053 | glutamine | Known | Amino acid |
| M00054 | tryptophan | Known | Amino acid |
| M00059 | histidine | Known | Amino acid |
| M00060 | leucine | Known | Amino acid |
| M00063 | cholesterol | Known | Lipid |
| M00064 | phenylalanine | Known | Amino acid |
| M00513 | creatinine | Known | Amino acid |
| M00527 | lactate | Known | Carbohydrate |
| M00542 | 3-hydroxybutyrate (BHBA) | Known | Lipid |
| M00553 | cotinine | Known | Xenobiotics |
| M00569 | caffeine | Known | Xenobiotics |
| M00575 | arabinose | Known | Carbohydrate |
| M00577 | fructose | Known | Carbohydrate |
| M00584 | mannose | Known | Carbohydrate |
| M00599 | pyruvate | Known | Carbohydrate |
| M00606 | uridine | Known | Nucleotide |
| M01105 | linoleate (18:2n6) | Known | Lipid |
| M01107 | allantoin | Known | Nucleotide |
| M01110 | arachidonate (20:4n6) | Known | Lipid |
| M01114 | deoxycholate | Known | Lipid |
| M01121 | margarate (17:0) | Known | Lipid |
| M01123 | inosine | Known | Nucleotide |
| M01125 | isoleucine | Known | Amino acid |
| M01284 | threonine | Known | Amino acid |
| M01299 | tyrosine | Known | Amino acid |
| M01301 | lysine | Known | Amino acid |
| M01302 | methionine | Known | Amino acid |
| M01303 | malate | Known | Energy |
| M01336 | palmitate (16:0) | Known | Lipid |
| M01356 | nonadecanoate (19:0) | Known | Lipid |
| M01358 | stearate (18:0) | Known | Lipid |
| M01359 | oleate (18:1n9) | Known | Lipid |
| M01361 | pentadecanoate (15:0) | Known | Lipid |
| M01365 | myristate (14:0) | Known | Lipid |
| M01444 | pipecolate | Known | Amino acid |
| M01493 | ornithine | Known | Amino acid |
| M01494 | 5-oxoproline | Known | Amino acid |
| M01508 | pantothenate | Known | Cofactors and vitamins |
| M01515 | salicylate | Known | Xenobiotics |
| M01558 | 4-acetamidobutanoate | Known | Amino acid |
| M01561 | alpha-tocopherol | Known | Cofactors and vitamins |
| M01564 | citrate | Known | Energy |
| M01572 | glycerate | Known | Carbohydrate |
| M01573 | guanosine | Known | Nucleotide |
| M01585 | N-acetylalanine | Known | Amino acid |
| M01604 | urate | Known | Nucleotide |
| M01605 | ursodeoxycholate | Known | Lipid |
| M01638 | arginine | Known | Amino acid |
| M01640 | ascorbate (Vitamin C) | Known | Cofactors and vitamins |
| M01644 | heptanoate (7:0) | Known | Lipid |
| M01645 | laurate (12:0) | Known | Lipid |
| M01649 | valine | Known | Amino acid |
| M01670 | urea | Known | Amino acid |
| M01712 | cortisol | Known | Lipid |
| M01769 | cortisone | Known | Lipid |
| M01898 | proline | Known | Amino acid |
| M02132 | citrulline | Known | Amino acid |
| M02137 | biliverdin | Known | Cofactors and vitamins |
| M02342 | serotonin (5HT) | Known | Amino acid |
| M02730 | gamma-glutamylglutamine | Known | Peptide |
| M02734 | gamma-glutamyltyrosine | Known | Peptide |
| M03127 | hypoxanthine | Known | Nucleotide |
| M03141 | betaine | Known | Amino acid |
| M03147 | xanthine | Known | Nucleotide |
| M10642 | caprate (10:0) | Known | Lipid |
| M11438 | phosphate | Known | Energy |
| M12017 | 3-methoxytyrosine | Known | Amino acid |
| M12035 | pelargonate (9:0) | Known | Lipid |
| M12067 | undecanoate (11:0) | Known | Lipid |
| M12129 | beta-hydroxyisovalerate | Known | Amino acid |
| M12261 | taurodeoxycholate | Known | Lipid |
| M12593 | X-02973 | Unknown |  |
| M12626 | X-03003 | Unknown |  |
| M12768 | X-03088 | Unknown |  |
| M12774 | X-03094 | Unknown |  |
| M15122 | glycerol | Known | Lipid |
| M15140 | kynurenine | Known | Amino acid |
| M15335 | mannitol | Known | Carbohydrate |
| M15365 | glycerol 3-phosphate (G3P) | Known | Lipid |
| M15488 | acetylphosphate | Known | Energy |
| M15500 | carnitine | Known | Lipid |
| M15506 | choline | Known | Lipid |
| M15630 | N-acetylornithine | Known | Amino acid |
| M15650 | N1-methyladenosine | Known | Nucleotide |
| M15676 | 3-methyl-2-oxovalerate | Known | Amino acid |
| M15677 | 3-methylhistidine | Known | Amino acid |
| M15749 | 3-phenylpropionate (hydrocinnamate) | Known | Amino acid |
| M15753 | hippurate | Known | Xenobiotics |
| M15778 | benzoate | Known | Xenobiotics |
| M15958 | phenylacetate | Known | Amino acid |
| M15990 | glycerophosphorylcholine (GPC) | Known | Lipid |
| M15996 | aspartate | Known | Amino acid |
| M16634 | X-04357 | Unknown |  |
| M16816 | X-04494 | Unknown |  |
| M16818 | X-04495 | Unknown |  |
| M16821 | X-04498 | Unknown |  |
| M16822 | X-04499--3,4-dihydroxybutyrate | Identified | Amino acid |
| M16823 | X-04500 | Unknown |  |
| M17799 | ibuprofen | Known | Xenobiotics |
| M17805 | dihomo-linoleate (20:2n6) | Known | Lipid |
| M17807 | X-18601 | Unknown |  |
| M17945 | 2-hydroxystearate | Known | Lipid |
| M18254 | paraxanthine | Known | Xenobiotics |
| M18281 | 2-hydroxyhippurate (salicylurate) | Known | Xenobiotics |
| M18283 | X-05426 | Unknown |  |
| M18335 | quinate | Known | Xenobiotics |
| M18349 | indolelactate | Known | Amino acid |
| M18357 | glycylvaline | Known | Peptide |
| M18369 | gamma-glutamylleucine | Known | Peptide |
| M18392 | theobromine | Known | Xenobiotics |
| M18394 | theophylline | Known | Xenobiotics |
| M18467 | eicosapentaenoate (EPA; 20:5n3) | Known | Lipid |
| M18474 | estrone 3-sulfate | Known | Lipid |
| M18476 | glycocholate | Known | Lipid |
| M18477 | glycodeoxycholate | Known | Lipid |
| M18494 | taurochenodeoxycholate | Known | Lipid |
| M18497 | taurocholate | Known | Lipid |
| M18929 | X-05907 | Unknown |  |
| M19323 | docosahexaenoate (DHA; 22:6n3) | Known | Lipid |
| M19324 | 1-stearoylglycerophosphoinositol | Known | Lipid |
| M19362 | X-06226 | Unknown |  |
| M19364 | X-06246 | Unknown |  |
| M19368 | X-06267 | Unknown |  |
| M19396 | X-06307 | Unknown |  |
| M19414 | X-06350 | Unknown |  |
| M19415 | X-06351 | Unknown |  |
| M19934 | myo-inositol | linoleat | Lipid |
| M20489 | glucose | Known | Carbohydrate |
| M20675 | 1,5-anhydroglucitol (1,5-AG) | Known | Carbohydrate |
| M20699 | erythritol | Known | Xenobiotics |
| M21044 | 2-hydroxybutyrate (AHB) | Known | Amino acid |
| M21047 | 3-methyl-2-oxobutyrate | Known | Amino acid |
| M21049 | 1,6-anhydroglucose | Known | Carbohydrate |
| M21127 | 1-palmitoylglycerol (1-monopalmitin) | Known | Lipid |
| M21151 | saccharin | Known | Xenobiotics |
| M21184 | 1-oleoylglycerol (1-monoolein) | Known | Lipid |
| M21188 | 1-stearoylglycerol (1-monostearin) | Known | Lipid |
| M21630 | X-08402 | Unknown |  |
| M22030 | 2-hydroxyisobutyrate | Known | Amino acid |
| M22032 | X-08766 | Unknown |  |
| M22116 | 4-methyl-2-oxopentanoate | Known | Amino acid |
| M22130 | phenyllactate (PLA) | Known | Amino acid |
| M22138 | homocitrulline | Known | Amino acid |
| M22175 | aspartylphenylalanine | Known | Peptide |
| M22177 | levulinate (4-oxovalerate) | Known | Amino acid |
| M22189 | palmitoylcarnitine | Known | Lipid |
| M22481 | X-08988 | Unknown |  |
| M22548 | X-09026 | Unknown |  |
| M22649 | X-09108 | Unknown |  |
| M22842 | cholate | Known | Lipid |
| M24074 | X-09706 | Unknown |  |
| M25459 | X-10395 | Unknown |  |
| M25599 | X-10429 | Unknown |  |
| M27256 | X-10500 | Unknown |  |
| M27273 | X-10506 | Unknown |  |
| M27278 | X-10510 | Unknown |  |
| M27447 | 1-linoleoylglycerol (1-monolinolein) | Known | Lipid |
| M27513 | indoleacetate | Known | Amino acid |
| M27531 | hyodeoxycholate | Known | Lipid |
| M27672 | 3-indoxyl sulfate | Known | Amino acid |
| M27710 | N-acetylglycine | Known | Amino acid |
| M27716 | bilirubin (Z,Z) | Known | Cofactors and vitamins |
| M27718 | creatine | Known | Amino acid |
| M27722 | erythrose | Known | Carbohydrate |
| M27728 | glycerol 2-phosphate | Known | Xenobiotics |
| M27738 | threonate | Known | Cofactors and vitamins |
| M30805 | X-10810 | Unknown |  |
| M31453 | cysteine | Known | Amino acid |
| M31522 | pyroglutamylglycine | Known | Peptide |
| M31536 | N-(2-furoyl)glycine | Known | Xenobiotics |
| M31548 | DSGEGDFXAEGGGVR* | Known | Peptide |
| M31555 | pyridoxate | Known | Cofactors and vitamins |
| M31591 | androsterone sulfate | Known | Lipid |
| M31787 | 3-carboxy-4-methyl-5-propyl-2-furanpropanoate (CMPF) | Known | Lipid |
| M32197 | 3-(4-hydroxyphenyl)lactate | Known | Amino acid |
| M32198 | acetylcarnitine | Known | Lipid |
| M32315 | serine | Known | Amino acid |
| M32319 | trans-4-hydroxyproline | Known | Amino acid |
| M32322 | glutamate | Known | Amino acid |
| M32328 | hexanoylcarnitine | Known | Lipid |
| M32338 | glycine | Known | Amino acid |
| M32339 | alanine | Known | Amino acid |
| M32346 | glycochenodeoxycholate | Known | Lipid |
| M32348 | 2-aminobutyrate | Known | Amino acid |
| M32379 | scyllo-inositol | Known | Lipid |
| M32388 | dodecanedioate | Known | Lipid |
| M32393 | gamma-glutamylvaline | Known | Peptide |
| M32405 | indolepropionate | Known | Amino acid |
| M32412 | butyrylcarnitine | Known | Lipid |
| M32418 | myristoleate (14:1n5) | Known | Lipid |
| M32425 | dehydroisoandrosterone sulfate (DHEA-S) | Known | Lipid |
| M32445 | 3-methylxanthine | Known | Xenobiotics |
| M32452 | propionylcarnitine | Known | Lipid |
| M32489 | caproate (6:0) | Known | Lipid |
| M32492 | caprylate (8:0) | Known | Lipid |
| M32497 | 10-undecenoate (11:1n1) | Known | Lipid |
| M32504 | docosapentaenoate (n3 DPA; 22:5n3) | Known | Lipid |
| M32518 | X-11204 | Unknown |  |
| M32549 | X-02269 | Unknown |  |
| M32553 | phenol sulfate | Known | Amino acid |
| M32557 | X-06126 | Unknown |  |
| M32560 | X-07765 | Unknown |  |
| M32564 | X-11247 | Unknown |  |
| M32578 | X-11261 | Unknown |  |
| M32586 | bilirubin (E,E)* | Known | Cofactors and vitamins |
| M32587 | X-02249 | Unknown |  |
| M32593 | heme* | Known | Cofactors and vitamins |
| M32616 | X-11299 | Unknown |  |
| M32632 | X-11315 | Unknown |  |
| M32634 | X-11317 | Unknown |  |
| M32635 | 1-linoleoylglycerophosphoethanolamine* | Known | Lipid |
| M32644 | X-11327 | Unknown |  |
| M32651 | X-11334 | Unknown |  |
| M32654 | 3-dehydrocarnitine* | Known | Lipid |
| M32672 | pyroglutamine* | Known | Amino acid |
| M32675 | C-glycosyltryptophan* | Known | Amino acid |
| M32691 | X-11374 | Unknown |  |
| M32698 | X-11381 | Unknown |  |
| M32709 | X-03056--N-[3-(2-Oxopyrrolidin-1-yl)propyl]acetamide | Identified | Amino acid |
| M32729 | X-11412 | Unknown |  |
| M32735 | X-01911 | Unknown |  |
| M32739 | X-11422--xanthine | Identified | Nucleotide |
| M32740 | X-11423--O-sulfo-L-tyrosine | Identified | Amino acid |
| M32753 | X-09789 | Unknown |  |
| M32754 | X-11437 | Unknown |  |
| M32755 | X-11438 | Unknown |  |
| M32757 | X-11440 | Unknown |  |
| M32758 | X-11441 | Unknown |  |
| M32759 | X-11442 | Unknown |  |
| M32761 | X-11444 | Unknown |  |
| M32762 | X-11445--5-alpha-pregnan-3beta,20alpha-disulfate | Identified | Lipid |
| M32769 | X-11452 | Unknown |  |
| M32786 | X-11469 | Unknown |  |
| M32787 | X-11470 | Unknown |  |
| M32795 | X-11478 | Unknown |  |
| M32800 | X-11483 | Unknown |  |
| M32802 | X-11485 | Unknown |  |
| M32808 | X-11491 | Unknown |  |
| M32814 | X-11497 | Unknown |  |
| M32836 | HWESASXX* | Known | Peptide |
| M32838 | X-11521 | Unknown |  |
| M32846 | X-11529 | Unknown |  |
| M32847 | X-11530 | Unknown |  |
| M32854 | X-11537 | Unknown |  |
| M32855 | X-11538 | Unknown |  |
| M32857 | X-11540 | Unknown |  |
| M32863 | X-11546 | Unknown |  |
| M32867 | X-11550 | Unknown |  |
| M32869 | X-11552 | Unknown |  |
| M32910 | X-11593--O-methylascorbate* | Identified | Cofactors and vitamins |
| M32980 | adrenate (22:4n6) | Known | Lipid |
| M33009 | homostachydrine* | Known | Xenobiotics |
| M33084 | ADSGEGDFXAEGGGVR* | Known | Peptide |
| M33131 | X-11786--methylcysteine | Identified | Amino acid |
| M33132 | X-11787 | Unknown |  |
| M33137 | X-11792 | Unknown |  |
| M33138 | X-11793--oxidized bilirubin* | Identified | Cofactors and vitamins |
| M33140 | X-11795 | Unknown |  |
| M33144 | X-11799 | Unknown |  |
| M33150 | X-11805 | Unknown |  |
| M33163 | X-11818 | Unknown |  |
| M33165 | X-11820 | Unknown |  |
| M33173 | 2-hydroxyacetaminophen sulfate* | Known | Xenobiotics |
| M33178 | 2-methoxyacetaminophen sulfate* | Known | Xenobiotics |
| M33188 | X-11843 | Unknown |  |
| M33190 | X-11845 | Unknown |  |
| M33192 | X-11847 | Unknown |  |
| M33194 | X-11849 | Unknown |  |
| M33195 | X-11850 | Unknown |  |
| M33197 | X-11852 | Unknown |  |
| M33203 | X-11858 | Unknown |  |
| M33204 | X-11859 | Unknown |  |
| M33221 | X-11876 | Unknown |  |
| M33228 | 1-arachidonoylglycerophosphocholine* | Known | Lipid |
| M33230 | 1-palmitoleoylglycerophosphocholine* | Known | Lipid |
| M33250 | X-11905 | Unknown |  |
| M33353 | X-12007 | Unknown |  |
| M33359 | X-12013 | Unknown |  |
| M33363 | gamma-glutamylmethionine* | Known | Peptide |
| M33364 | gamma-glutamylthreonine* | Known | Peptide |
| M33380 | X-12029 | Unknown |  |
| M33384 | salicyluric glucuronide* | Known | Xenobiotics |
| M33389 | X-12038 | Unknown |  |
| M33390 | X-12039 | Unknown |  |
| M33391 | X-12040 | Unknown |  |
| M33408 | X-12056 | Unknown |  |
| M33415 | X-12063 | Unknown |  |
| M33420 | gamma-tocopherol | Known | Cofactors and vitamins |
| M33422 | gamma-glutamylphenylalanine | Known | Peptide |
| M33423 | p-acetamidophenylglucuronide | Known | Xenobiotics |
| M33441 | isobutyrylcarnitine | Known | Amino acid |
| M33442 | pseudouridine | Known | Nucleotide |
| M33443 | valerate | Known | Lipid |
| M33447 | palmitoleate (16:1n7) | Known | Lipid |
| M33453 | alpha-ketoglutarate | Known | Energy |
| M33477 | erythronate* | Known | Carbohydrate |
| M33488 | lathosterol | Known | Lipid |
| M33507 | X-12092 | Unknown |  |
| M33508 | X-12093 | Unknown |  |
| M33509 | X-12094 | Unknown |  |
| M33510 | X-12095--N1-methyl-3-pyridone-4-carboxamide | Identified | Nucleotide |
| M33515 | X-12100--hydroxytryptophan* | Identified | Amino acid |
| M33531 | X-12116 | Unknown |  |
| M33587 | eicosenoate (20:1n9 or 11) | Known | Lipid |
| M33609 | X-12188 | Unknown |  |
| M33610 | X-12189 | Unknown |  |
| M33627 | X-12206 | Unknown |  |
| M33633 | X-12212 | Unknown |  |
| M33637 | X-12216 | Unknown |  |
| M33638 | X-12217 | Unknown |  |
| M33652 | X-12230 | Unknown |  |
| M33653 | X-12231 | Unknown |  |
| M33658 | X-12236 | Unknown |  |
| M33666 | X-12244--N-acetylcarnosine | Identified | Peptide |
| M33675 | X-12253 | Unknown |  |
| M33683 | X-12261 | Unknown |  |
| M33751 | X-12329 | Unknown |  |
| M33782 | X-10346 | Unknown |  |
| M33801 | ADpSGEGDFXAEGGGVR* | Known | Peptide |
| M33821 | 1-eicosatrienoylglycerophosphocholine* | Known | Lipid |
| M33822 | 1-docosahexaenoylglycerophosphocholine* | Known | Lipid |
| M33833 | X-12405 | Unknown |  |
| M33835 | X-12407 | Unknown |  |
| M33864 | X-12428 | Unknown |  |
| M33871 | 1-eicosadienoylglycerophosphocholine* | Known | Lipid |
| M33877 | X-12435 | Unknown |  |
| M33883 | X-12441--12-hydroxyeicosatetraenoate (12-HETE) | Identified | Lipid |
| M33884 | X-12442--5,8-tetradecadienoate | Identified | Lipid |
| M33885 | X-12443 | Unknown |  |
| M33892 | X-12450 | Unknown |  |
| M33901 | X-12456 | Unknown |  |
| M33910 | X-12465 | Unknown |  |
| M33935 | piperine | Known | Xenobiotics |
| M33936 | octanoylcarnitine | Known | Lipid |
| M33937 | alpha-hydroxyisovalerate | Known | Amino acid |
| M33939 | N-acetylthreonine | Known | Amino acid |
| M33941 | decanoylcarnitine | Known | Lipid |
| M33955 | 1-palmitoylglycerophosphocholine | Known | Lipid |
| M33957 | 1-heptadecanoylglycerophosphocholine | Known | Lipid |
| M33960 | 1-oleoylglycerophosphocholine | Known | Lipid |
| M33961 | 1-stearoylglycerophosphocholine | Known | Lipid |
| M33968 | 5-dodecenoate (12:1n7) | Known | Lipid |
| M33969 | stearidonate (18:4n3) | Known | Lipid |
| M33971 | 10-heptadecenoate (17:1n7) | Known | Lipid |
| M33972 | 10-nonadecenoate (19:1n9) | Known | Lipid |
| M33973 | epiandrosterone sulfate | Known | Lipid |
| M34035 | linolenate [alpha or gamma; (18:3n3 or 6)] | Known | Lipid |
| M34040 | X-12510--2-aminooctanoic acid | Identified | Amino acid |
| M34062 | X-12524 | Unknown |  |
| M34106 | bilirubin (E,Z or Z,E)* | Known | Cofactors and vitamins |
| M34109 | metoprolol acid metabolite* | Known | Xenobiotics |
| M34112 | X-12544 | Unknown |  |
| M34123 | X-12556 | Unknown |  |
| M34214 | 1-arachidonoylglycerophosphoinositol* | Known | Lipid |
| M34221 | X-12627 | Unknown |  |
| M34244 | X-12644 | Unknown |  |
| M34245 | X-12645 | Unknown |  |
| M34283 | asparagine | Known | Amino acid |
| M34289 | X-12680 | Unknown |  |
| M34306 | X-12696 | Unknown |  |
| M34314 | X-12704 | Unknown |  |
| M34322 | X-12712 | Unknown |  |
| M34327 | X-12717 | Unknown |  |
| M34329 | X-12719 | Unknown |  |
| M34336 | X-12726 | Unknown |  |
| M34338 | X-12728 | Unknown |  |
| M34339 | X-12729 | Unknown |  |
| M34344 | X-12734 | Unknown |  |
| M34350 | X-12740 | Unknown |  |
| M34359 | X-12749 | Unknown |  |
| M34365 | 3-(cystein-S-yl)acetaminophen* | Known | Xenobiotics |
| M34384 | stachydrine | Known | Xenobiotics |
| M34389 | 1-methylxanthine | Known | Xenobiotics |
| M34390 | 7-methylxanthine | Known | Xenobiotics |
| M34395 | 1-methylurate | Known | Xenobiotics |
| M34400 | 1,7-dimethylurate | Known | Xenobiotics |
| M34404 | 1,3,7-trimethylurate | Known | Xenobiotics |
| M34407 | isovalerylcarnitine | Known | Amino acid |
| M34409 | stearoylcarnitine | Known | Lipid |
| M34416 | 1-stearoylglycerophosphoethanolamine | Known | Lipid |
| M34419 | 1-linoleoylglycerophosphocholine | Known | Lipid |
| M34420 | bradykinin, des-arg(9) | Known | Peptide |
| M34441 | X-12771 | Unknown |  |
| M34453 | X-12776 | Unknown |  |
| M34456 | gamma-glutamylisoleucine* | Known | Peptide |
| M34469 | X-12786 | Unknown |  |
| M34481 | X-12798 | Unknown |  |
| M34499 | X-12816 | Unknown |  |
| M34513 | X-12830 | Unknown |  |
| M34516 | X-12833 | Unknown |  |
| M34527 | X-12844 | Unknown |  |
| M34530 | X-12847 | Unknown |  |
| M34533 | X-12850 | Unknown |  |
| M34534 | laurylcarnitine | Known | Lipid |
| M34535 | X-12851 | Unknown |  |
| M34539 | X-12855 | Unknown |  |
| M34674 | X-12990--docosapentaenoic acid (n6-DPA) | Identified | Lipid |
| M34732 | isovalerate | Known | Lipid |
| M34761 | X-13069 | Unknown |  |
| M34878 | X-13183--stearamide | Identified | Lipid |
| M34912 | X-13215 | Unknown |  |
| M35114 | 7-methylguanine | Known | Nucleotide |
| M35126 | phenylacetylglutamine | Known | Amino acid |
| M35127 | pro-hydroxy-pro | Known | Peptide |
| M35137 | N2,N2-dimethylguanosine | Known | Nucleotide |
| M35159 | cysteine-glutathione disulfide | Known | Amino acid |
| M35160 | oleoylcarnitine | Known | Lipid |
| M35186 | 1-arachidonoylglycerophosphoethanolamine* | Known | Lipid |
| M35187 | X-13429 | Unknown |  |
| M35189 | X-13431--nonanoylcarnitine* | Identified | Lipid |
| M35193 | X-13435 | Unknown |  |
| M35240 | X-13477 | Unknown |  |
| M35253 | 2-palmitoylglycerophosphocholine* | Known | Lipid |
| M35254 | 2-oleoylglycerophosphocholine* | Known | Lipid |
| M35255 | 2-stearoylglycerophosphocholine* | Known | Lipid |
| M35257 | 2-linoleoylglycerophosphocholine* | Known | Lipid |
| M35270 | X-13496 | Unknown |  |
| M35305 | 1-palmitoylglycerophosphoinositol* | Known | Lipid |
| M35320 | catechol sulfate | Known | Xenobiotics |
| M35322 | hydroquinone sulfate | Known | Xenobiotics |
| M35326 | X-13548 | Unknown |  |
| M35327 | X-13549 | Unknown |  |
| M35331 | X-13553 | Unknown |  |
| M35397 | X-13619 | Unknown |  |
| M35431 | 2-methylbutyroylcarnitine | Known | Amino acid |
| M35433 | hydroxyisovaleroyl carnitine | Known | Amino acid |
| M35439 | glutaroyl carnitine | Known | Amino acid |
| M35451 | X-13658 | Unknown |  |
| M35464 | X-13671 | Unknown |  |
| M35472 | 2-tetradecenoyl carnitine | Known | Lipid |
| M35527 | 4-hydroxyhippurate | Known | Xenobiotics |
| M35551 | X-13741 | Unknown |  |
| M35626 | 1-myristoylglycerophosphocholine | Known | Lipid |
| M35628 | 1-oleoylglycerophosphoethanolamine | Known | Lipid |
| M35631 | 1-palmitoylglycerophosphoethanolamine | Known | Lipid |
| M35635 | 3-(3-hydroxyphenyl)propionate | Known | Amino acid |
| M35669 | tetradecanedioate | Known | Lipid |
| M35675 | 2-hydroxypalmitate | Known | Lipid |
| M35678 | hexadecanedioate | Known | Lipid |
| M35718 | dihomo-linolenate (20:3n3 or n6) | Known | Lipid |
| M35754 | X-13859 | Unknown |  |
| M35854 | threitol | Known | Carbohydrate |
| M35977 | X-14056 | Unknown |  |
| M35978 | X-14057 | Unknown |  |
| M36009 | X-14086 | Unknown |  |
| M36095 | thymol sulfate | Known | Xenobiotics |
| M36097 | 4-acetaminophen sulfate | Known | Xenobiotics |
| M36098 | 4-vinylphenol sulfate | Known | Xenobiotics |
| M36099 | 4-ethylphenylsulfate | Known | Xenobiotics |
| M36103 | p-cresol sulfate | Known | Amino acid |
| M36115 | X-14189--leucylalanine | Identified | Peptide |
| M36131 | X-14205--alpha-glutamyltyrosine | Identified | Peptide |
| M36134 | X-14208--phenylalanylserine | Identified | Peptide |
| M36230 | X-14304--leucylalanine | Identified | Peptide |
| M36300 | X-14374 | Unknown |  |
| M36376 | X-14450--phenylalanylleucine | Identified | Peptide |
| M36399 | X-14473 | Unknown |  |
| M36468 | X-14541 | Unknown |  |
| M36515 | X-14588 | Unknown |  |
| M36552 | X-14625 | Unknown |  |
| M36553 | X-14626 | Unknown |  |
| M36559 | X-14632 | Unknown |  |
| M36585 | X-14658 | Unknown |  |
| M36589 | X-14662 | Unknown |  |
| M36673 | X-14745 | Unknown |  |
| M36738 | gamma-glutamylglutamate | Known | Peptide |
| M36754 | octadecanedioate | Known | Lipid |
| M36756 | leucylleucine | Known | Peptide |
| M36776 | 7-alpha-hydroxy-3-oxo-4-cholestenoate (7-Hoca) | Known | Lipid |
| M36802 | n-Butyl Oleate | Known | Lipid |
| M36808 | dimethylarginine (SDMA + ADMA) | Known | Amino acid |
| M36850 | taurolithocholate 3-sulfate | Known | Lipid |
| M37004 | X-14977--vanillin | Identified | Xenobiotics |
| M37058 | succinylcarnitine | Known | Energy |
| M37097 | tryptophan betaine | Known | Amino acid |
| M37104 | cyclo(leu-pro) | Known | Peptide |
| M37112 | chiro-inositol | Known | Lipid |
| M37190 | 5alpha-androstan-3beta,17beta-diol disulfate | Known | Lipid |
| M37202 | 4-androsten-3beta,17beta-diol disulfate 1* | Known | Lipid |
| M37203 | 4-androsten-3beta,17beta-diol disulfate 2* | Known | Lipid |
| M37253 | 2-hydroxyglutarate | Known | Lipid |
| M37459 | ergothioneine | Known | Xenobiotics |
| M37506 | palmitoyl sphingomyelin | Known | Lipid |
| M38150 | phenylalanylphenylalanine | Known | Peptide |
| M38178 | cis-4-decenoyl carnitine | Known | Lipid |
| M38768 | 15-methylpalmitate (isobar with 2-methylpalmitate) | Known | Lipid |

**Supplemental table 3. Summary information on the SNPs used as instrumental variables.**

| **Metabolite ID** | **Metabolites** | **SNP** | **Effect Allele** | **Reference Allele** | **Summary statistics for metabolites** | | | **Summary statistics for SSNHL** | | |  |  | **Steiger direction** | **Steiger *P-*value** |
| --- | --- | --- | --- | --- | --- | --- | --- | --- | --- | --- | --- | --- | --- | --- |
|  |  |  |  |  | **Beta** | **SE** | ***P* value** | **Beta** | **SE** | ***P* value** | **R^2^** | **F** |  |  |
| M00063 | cholesterol | rs10011091 | T | C | 0.0086 | 0.0018 | 2.15E-06 | 0.0473 | 0.0390 | 2.26E-01 | 0.0031 | 22.82 | TRUE | 5.18E-06 |
|  |  | rs1005596 | A | G | 0.0115 | 0.0025 | 4.20E-06 | 0.0947 | 0.0580 | 1.03E-01 | 0.0029 | 21.15 | TRUE | 1.54E-05 |
|  |  | rs10207315 | T | G | 0.0078 | 0.0017 | 4.79E-06 | 0.0024 | 0.0332 | 9.41E-01 | 0.0029 | 21.05 | TRUE | 5.90E-06 |
|  |  | rs10493549 | A | G | -0.0090 | 0.0017 | 2.27E-07 | -0.0292 | 0.0333 | 3.81E-01 | 0.0038 | 28.02 | TRUE | 3.14E-07 |
|  |  | rs1077834 | T | C | -0.0113 | 0.0023 | 6.87E-07 | 0.0406 | 0.0293 | 1.66E-01 | 0.0033 | 24.13 | TRUE | 3.06E-06 |
|  |  | rs11022174 | A | G | 0.0101 | 0.0023 | 7.36E-06 | -0.0140 | 0.0267 | 6.01E-01 | 0.0026 | 19.28 | TRUE | 1.94E-05 |
|  |  | rs11106131 | T | G | -0.0089 | 0.0019 | 1.81E-06 | -0.0119 | 0.0314 | 7.05E-01 | 0.0030 | 21.94 | TRUE | 4.61E-06 |
|  |  | rs11111293 | T | C | -0.0080 | 0.0018 | 6.12E-06 | 0.0331 | 0.0306 | 2.80E-01 | 0.0027 | 19.75 | TRUE | 2.17E-05 |
|  |  | rs1124515 | A | G | -0.0078 | 0.0017 | 6.01E-06 | -0.0653 | 0.0290 | 2.44E-02 | 0.0029 | 21.05 | TRUE | 2.40E-05 |
|  |  | rs11610936 | T | C | -0.0121 | 0.0025 | 1.34E-06 | -0.0257 | 0.0442 | 5.61E-01 | 0.0032 | 23.42 | TRUE | 2.51E-06 |
|  |  | rs11813145 | A | G | 0.0076 | 0.0017 | 5.87E-06 | 0.0058 | 0.0281 | 8.38E-01 | 0.0027 | 19.98 | TRUE | 1.11E-05 |
|  |  | rs12648765 | A | G | 0.0564 | 0.0122 | 3.57E-06 | 0.0091 | 0.1311 | 9.45E-01 | 0.0120 | 21.35 | TRUE | 4.30E-06 |
|  |  | rs12881791 | T | G | -0.0083 | 0.0018 | 5.91E-06 | 0.0357 | 0.0279 | 2.00E-01 | 0.0029 | 21.26 | TRUE | 1.17E-05 |
|  |  | rs12896813 | T | C | -0.0082 | 0.0018 | 3.52E-06 | -0.0463 | 0.0343 | 1.77E-01 | 0.0028 | 20.75 | TRUE | 1.57E-05 |
|  |  | rs1433904 | T | C | 0.0442 | 0.0097 | 5.06E-06 | 0.1077 | 0.0951 | 2.58E-01 | 0.0116 | 20.74 | TRUE | 8.30E-06 |
|  |  | rs1479402 | T | C | 0.0306 | 0.0068 | 6.85E-06 | -0.0760 | 0.1742 | 6.62E-01 | 0.0027 | 20.24 | TRUE | 1.12E-05 |
|  |  | rs17182784 | T | C | -0.0082 | 0.0017 | 2.10E-06 | -0.0154 | 0.0273 | 5.74E-01 | 0.0031 | 23.26 | TRUE | 2.68E-06 |
|  |  | rs17293443 | T | C | 0.0078 | 0.0017 | 8.09E-06 | 0.0098 | 0.0290 | 7.34E-01 | 0.0029 | 21.05 | TRUE | 7.04E-06 |
|  |  | rs17683567 | T | C | 0.0078 | 0.0017 | 7.57E-06 | 0.0325 | 0.0328 | 3.21E-01 | 0.0029 | 21.05 | TRUE | 1.08E-05 |
|  |  | rs17728920 | A | C | 0.0081 | 0.0018 | 5.30E-06 | 0.0099 | 0.0362 | 7.85E-01 | 0.0027 | 20.24 | TRUE | 1.01E-05 |
|  |  | rs17765253 | A | G | 0.0096 | 0.0018 | 8.27E-08 | -0.0276 | 0.0303 | 3.61E-01 | 0.0038 | 28.44 | TRUE | 2.63E-07 |
|  |  | rs2026590 | T | G | 0.0080 | 0.0017 | 3.76E-06 | -0.0012 | 0.0316 | 9.70E-01 | 0.0030 | 22.14 | TRUE | 3.30E-06 |
|  |  | rs2050223 | A | G | -0.0078 | 0.0017 | 7.22E-06 | 0.0147 | 0.0327 | 6.54E-01 | 0.0029 | 21.05 | TRUE | 7.56E-06 |
|  |  | rs2130712 | A | C | 0.0165 | 0.0032 | 2.43E-07 | -0.0773 | 0.0583 | 1.85E-01 | 0.0036 | 26.58 | TRUE | 8.86E-07 |
|  |  | rs2259571 | T | G | -0.0078 | 0.0017 | 2.86E-06 | 0.0109 | 0.0275 | 6.92E-01 | 0.0029 | 21.05 | TRUE | 7.30E-06 |
|  |  | rs2407893 | A | G | 0.0079 | 0.0016 | 1.29E-06 | 0.0068 | 0.0261 | 7.93E-01 | 0.0033 | 24.37 | TRUE | 1.24E-06 |
|  |  | rs2869804 | A | G | 0.0078 | 0.0017 | 6.50E-06 | 0.0164 | 0.0312 | 6.00E-01 | 0.0029 | 21.05 | TRUE | 7.95E-06 |
|  |  | rs2870664 | T | C | 0.0147 | 0.0031 | 1.60E-06 | 0.0301 | 0.0267 | 2.60E-01 | 0.0126 | 22.46 | TRUE | 3.46E-06 |
|  |  | rs3747924 | T | C | 0.0083 | 0.0018 | 4.24E-06 | 0.0108 | 0.0328 | 7.42E-01 | 0.0029 | 21.26 | TRUE | 6.28E-06 |
|  |  | rs3802195 | T | G | 0.0086 | 0.0018 | 1.13E-06 | 0.0284 | 0.0368 | 4.41E-01 | 0.0031 | 22.82 | TRUE | 3.85E-06 |
|  |  | rs3897960 | A | T | -0.0084 | 0.0017 | 1.29E-06 | -0.0074 | 0.0333 | 8.23E-01 | 0.0033 | 24.41 | TRUE | 1.19E-06 |
|  |  | rs4401492 | T | G | 0.0082 | 0.0018 | 4.52E-06 | -0.0303 | 0.0347 | 3.83E-01 | 0.0028 | 20.75 | TRUE | 1.16E-05 |
|  |  | rs445925 | A | G | -0.0250 | 0.0040 | 4.11E-10 | -0.1009 | 0.0487 | 3.82E-02 | 0.0069 | 39.05 | TRUE | 2.84E-09 |
|  |  | rs4602115 | T | G | 0.0146 | 0.0033 | 7.65E-06 | 0.0431 | 0.0288 | 1.34E-01 | 0.0110 | 19.55 | TRUE | 1.70E-05 |
|  |  | rs4941743 | T | C | 0.0087 | 0.0017 | 3.28E-07 | -0.0011 | 0.0291 | 9.70E-01 | 0.0035 | 26.18 | TRUE | 4.23E-07 |
|  |  | rs602633 | T | G | -0.0102 | 0.0017 | 5.02E-09 | 0.0060 | 0.0310 | 8.47E-01 | 0.0049 | 35.99 | TRUE | 3.46E-09 |
|  |  | rs6438950 | C | G | 0.0131 | 0.0025 | 1.54E-07 | 0.0232 | 0.0386 | 5.49E-01 | 0.0037 | 27.45 | TRUE | 3.39E-07 |
|  |  | rs6511720 | T | G | -0.0136 | 0.0027 | 3.69E-07 | -0.0413 | 0.0423 | 3.30E-01 | 0.0034 | 25.36 | TRUE | 1.25E-06 |
|  |  | rs6573766 | T | G | -0.0085 | 0.0018 | 1.55E-06 | -0.0311 | 0.0290 | 2.84E-01 | 0.0030 | 22.29 | TRUE | 6.12E-06 |
|  |  | rs6603141 | A | G | -0.0081 | 0.0017 | 2.61E-06 | -0.0146 | 0.0304 | 6.31E-01 | 0.0031 | 22.70 | TRUE | 3.36E-06 |
|  |  | rs6970076 | T | C | -0.0079 | 0.0017 | 4.37E-06 | -0.0037 | 0.0285 | 8.97E-01 | 0.0029 | 21.59 | TRUE | 4.65E-06 |
|  |  | rs7595385 | T | C | 0.0237 | 0.0051 | 3.83E-06 | 0.0320 | 0.0605 | 5.97E-01 | 0.0029 | 21.59 | TRUE | 6.07E-06 |
|  |  | rs810551 | C | G | 0.0080 | 0.0017 | 4.66E-06 | 0.0041 | 0.0305 | 8.94E-01 | 0.0030 | 22.14 | TRUE | 3.52E-06 |
|  |  | rs871748 | T | C | -0.0082 | 0.0018 | 8.56E-06 | -0.0067 | 0.0319 | 8.34E-01 | 0.0028 | 20.75 | TRUE | 7.52E-06 |
|  |  | rs886282 | T | C | 0.0081 | 0.0018 | 3.71E-06 | -0.0139 | 0.0307 | 6.50E-01 | 0.0027 | 20.24 | TRUE | 1.14E-05 |
|  |  | rs927961 | T | G | -0.0079 | 0.0017 | 5.06E-06 | 0.0166 | 0.0328 | 6.12E-01 | 0.0029 | 21.59 | TRUE | 5.98E-06 |
| M01358 | stearate(18:0) | rs10021069 | T | C | 0.0238 | 0.0050 | 1.81E-06 | 0.0941 | 0.0939 | 3.16E-01 | 0.0031 | 22.65 | TRUE | 4.89E-06 |
|  |  | rs10152773 | T | C | -0.0224 | 0.0049 | 4.73E-06 | -0.0046 | 0.0752 | 9.51E-01 | 0.0028 | 20.89 | TRUE | 6.33E-06 |
|  |  | rs10160169 | A | G | 0.0084 | 0.0018 | 3.78E-06 | 0.0139 | 0.0265 | 6.00E-01 | 0.0030 | 21.77 | TRUE | 5.51E-06 |
|  |  | rs10242171 | A | G | -0.0120 | 0.0026 | 4.25E-06 | 0.0350 | 0.0414 | 3.98E-01 | 0.0029 | 21.30 | TRUE | 8.65E-06 |
|  |  | rs10498606 | A | C | -0.0077 | 0.0017 | 6.40E-06 | 0.0121 | 0.0257 | 6.38E-01 | 0.0028 | 20.51 | TRUE | 1.00E-05 |
|  |  | rs1075227 | T | C | 0.0090 | 0.0020 | 6.80E-06 | 0.0014 | 0.0260 | 9.58E-01 | 0.0036 | 20.24 | TRUE | 8.33E-06 |
|  |  | rs10757839 | T | C | 0.0079 | 0.0017 | 4.74E-06 | -0.0335 | 0.0259 | 1.95E-01 | 0.0029 | 21.59 | TRUE | 1.00E-05 |
|  |  | rs10853932 | T | C | 0.0080 | 0.0018 | 5.45E-06 | -0.0069 | 0.0271 | 7.99E-01 | 0.0027 | 19.75 | TRUE | 1.29E-05 |
|  |  | rs11118120 | A | C | -0.0121 | 0.0025 | 1.18E-06 | -0.0095 | 0.0322 | 7.68E-01 | 0.0032 | 23.42 | TRUE | 2.06E-06 |
|  |  | rs12353451 | A | G | -0.0223 | 0.0049 | 6.06E-06 | 0.0174 | 0.0834 | 8.34E-01 | 0.0028 | 20.71 | TRUE | 7.67E-06 |
|  |  | rs1237044 | T | C | 0.0081 | 0.0018 | 5.10E-06 | 0.0034 | 0.0259 | 8.97E-01 | 0.0027 | 20.24 | TRUE | 9.21E-06 |
|  |  | rs13355979 | A | G | -0.0076 | 0.0017 | 6.23E-06 | 0.0066 | 0.0261 | 8.01E-01 | 0.0027 | 19.98 | TRUE | 1.14E-05 |
|  |  | rs1348656 | T | C | -0.0137 | 0.0024 | 1.17E-08 | 0.0209 | 0.0329 | 5.26E-01 | 0.0044 | 32.58 | TRUE | 2.73E-08 |
|  |  | rs1426441 | A | G | 0.0113 | 0.0025 | 4.87E-06 | 0.0339 | 0.0324 | 2.95E-01 | 0.0028 | 20.42 | TRUE | 1.52E-05 |
|  |  | rs1477025 | T | C | 0.0087 | 0.0017 | 4.83E-07 | -0.0315 | 0.0262 | 2.29E-01 | 0.0035 | 26.18 | TRUE | 9.84E-07 |
|  |  | rs1662999 | T | C | 0.0079 | 0.0017 | 4.21E-06 | -0.0187 | 0.0258 | 4.70E-01 | 0.0029 | 21.59 | TRUE | 6.89E-06 |
|  |  | rs1922419 | A | G | -0.0182 | 0.0039 | 3.37E-06 | 0.0339 | 0.0325 | 2.96E-01 | 0.0122 | 21.75 | TRUE | 4.82E-06 |
|  |  | rs2100884 | T | C | -0.0084 | 0.0017 | 8.60E-07 | 0.0316 | 0.0256 | 2.17E-01 | 0.0033 | 24.41 | TRUE | 2.41E-06 |
|  |  | rs2115366 | T | C | -0.0079 | 0.0017 | 3.93E-06 | 0.0024 | 0.0277 | 9.31E-01 | 0.0029 | 21.59 | TRUE | 4.51E-06 |
|  |  | rs216283 | A | C | 0.0080 | 0.0017 | 2.63E-06 | -0.0382 | 0.0256 | 1.36E-01 | 0.0030 | 22.14 | TRUE | 8.69E-06 |
|  |  | rs2280248 | A | G | -0.0077 | 0.0017 | 5.55E-06 | -0.0195 | 0.0257 | 4.47E-01 | 0.0028 | 20.51 | TRUE | 1.21E-05 |
|  |  | rs2489020 | T | C | -0.0081 | 0.0017 | 2.30E-06 | -0.0387 | 0.0256 | 1.31E-01 | 0.0031 | 22.70 | TRUE | 6.71E-06 |
|  |  | rs281216 | T | G | 0.0149 | 0.0033 | 8.26E-06 | 0.0235 | 0.0407 | 5.63E-01 | 0.0028 | 20.38 | TRUE | 1.15E-05 |
|  |  | rs3087751 | A | C | 0.0078 | 0.0017 | 5.23E-06 | 0.0035 | 0.0267 | 8.95E-01 | 0.0029 | 21.05 | TRUE | 6.13E-06 |
|  |  | rs3748771 | T | G | -0.0671 | 0.0149 | 6.35E-06 | 0.0758 | 0.1646 | 6.45E-01 | 0.0114 | 20.26 | TRUE | 8.56E-06 |
|  |  | rs3793900 | A | C | -0.0078 | 0.0017 | 5.73E-06 | 0.0172 | 0.0284 | 5.44E-01 | 0.0029 | 21.05 | TRUE | 8.38E-06 |
|  |  | rs3863373 | A | C | -0.0080 | 0.0017 | 4.21E-06 | -0.0187 | 0.0268 | 4.85E-01 | 0.0030 | 22.14 | TRUE | 5.15E-06 |
|  |  | rs4420815 | A | G | -0.0140 | 0.0030 | 3.06E-06 | -0.0158 | 0.0328 | 6.30E-01 | 0.0039 | 21.77 | TRUE | 4.90E-06 |
|  |  | rs4507103 | A | G | 0.0077 | 0.0017 | 6.89E-06 | 0.0001 | 0.0259 | 9.97E-01 | 0.0028 | 20.51 | TRUE | 7.41E-06 |
|  |  | rs4710948 | A | G | -0.0085 | 0.0017 | 6.94E-07 | 0.0029 | 0.0264 | 9.11E-01 | 0.0034 | 24.99 | TRUE | 8.16E-07 |
|  |  | rs4750059 | C | G | -0.0134 | 0.0030 | 6.49E-06 | 0.0032 | 0.0286 | 9.11E-01 | 0.0027 | 19.95 | TRUE | 1.06E-05 |
|  |  | rs603424 | A | G | 0.0225 | 0.0024 | 6.06E-21 | -0.0728 | 0.0402 | 6.96E-02 | 0.0118 | 87.87 | TRUE | 2.09E-19 |
|  |  | rs6733405 | A | G | -0.0082 | 0.0017 | 2.42E-06 | 0.0170 | 0.0257 | 5.07E-01 | 0.0032 | 23.26 | TRUE | 2.87E-06 |
|  |  | rs6883481 | T | C | 0.0077 | 0.0017 | 6.17E-06 | -0.0133 | 0.0262 | 6.12E-01 | 0.0028 | 20.51 | TRUE | 1.03E-05 |
|  |  | rs727892 | A | G | 0.0083 | 0.0017 | 8.15E-07 | -0.0231 | 0.0256 | 3.67E-01 | 0.0032 | 23.83 | TRUE | 2.55E-06 |
|  |  | rs7583745 | T | C | -0.0262 | 0.0055 | 1.49E-06 | 0.0333 | 0.0467 | 4.76E-01 | 0.0127 | 22.67 | TRUE | 2.71E-06 |
|  |  | rs7681360 | A | G | 0.0078 | 0.0017 | 4.19E-06 | -0.0062 | 0.0256 | 8.09E-01 | 0.0029 | 21.05 | TRUE | 6.60E-06 |
|  |  | rs7996008 | A | G | 0.0079 | 0.0017 | 5.69E-06 | -0.0374 | 0.0270 | 1.67E-01 | 0.0029 | 21.59 | TRUE | 1.06E-05 |
|  |  | rs954820 | A | G | 0.0083 | 0.0018 | 2.57E-06 | -0.0265 | 0.0261 | 3.10E-01 | 0.0029 | 21.26 | TRUE | 9.86E-06 |
|  |  | rs9568232 | T | C | 0.0126 | 0.0028 | 5.06E-06 | -0.0301 | 0.0438 | 4.91E-01 | 0.0027 | 20.24 | TRUE | 1.32E-05 |
|  |  | rs9809122 | A | G | -0.0087 | 0.0017 | 3.92E-07 | -0.0039 | 0.0266 | 8.84E-01 | 0.0035 | 26.18 | TRUE | 4.58E-07 |
| M01508 | pantothenate | rs10919047 | A | C | 0.0176 | 0.0035 | 6.75E-07 | 0.0455 | 0.0438 | 2.98E-01 | 0.0035 | 25.28 | TRUE | 1.35E-06 |
|  |  | rs11719633 | A | G | -0.0206 | 0.0044 | 3.01E-06 | 0.0012 | 0.0526 | 9.82E-01 | 0.0031 | 21.91 | TRUE | 3.64E-06 |
|  |  | rs12544560 | A | G | 0.0164 | 0.0035 | 3.32E-06 | -0.0990 | 0.0437 | 2.34E-02 | 0.0031 | 21.95 | TRUE | 1.53E-05 |
|  |  | rs13212846 | A | C | -0.0241 | 0.0052 | 2.88E-06 | 0.0218 | 0.0638 | 7.33E-01 | 0.0030 | 21.47 | TRUE | 5.63E-06 |
|  |  | rs1350858 | A | C | 0.0152 | 0.0034 | 8.21E-06 | 0.0285 | 0.0368 | 4.38E-01 | 0.0028 | 19.98 | TRUE | 1.58E-05 |
|  |  | rs1395 | A | G | 0.0172 | 0.0027 | 8.14E-11 | 0.0086 | 0.0279 | 7.57E-01 | 0.0056 | 40.57 | TRUE | 3.86E-10 |
|  |  | rs17049936 | T | C | -0.0187 | 0.0042 | 9.58E-06 | 0.0425 | 0.0339 | 2.10E-01 | 0.0028 | 19.82 | TRUE | 2.30E-05 |
|  |  | rs17082600 | T | C | 0.0476 | 0.0094 | 4.08E-07 | 0.0220 | 0.1522 | 8.85E-01 | 0.0036 | 25.64 | TRUE | 5.99E-07 |
|  |  | rs17751805 | A | G | 0.0628 | 0.0135 | 3.40E-06 | -0.0896 | 0.0638 | 1.60E-01 | 0.0129 | 21.61 | TRUE | 5.74E-06 |
|  |  | rs1975271 | T | C | 0.0116 | 0.0026 | 8.68E-06 | -0.0161 | 0.0259 | 5.34E-01 | 0.0028 | 19.90 | TRUE | 1.49E-05 |
|  |  | rs2173615 | T | G | 0.0125 | 0.0026 | 1.69E-06 | 0.0096 | 0.0262 | 7.14E-01 | 0.0032 | 23.11 | TRUE | 2.51E-06 |
|  |  | rs2526477 | A | G | -0.0120 | 0.0026 | 2.99E-06 | -0.0207 | 0.0256 | 4.19E-01 | 0.0030 | 21.30 | TRUE | 8.34E-06 |
|  |  | rs271072 | A | C | -0.0118 | 0.0026 | 5.94E-06 | -0.0178 | 0.0288 | 5.36E-01 | 0.0029 | 20.59 | TRUE | 1.05E-05 |
|  |  | rs2967437 | A | G | -0.0126 | 0.0027 | 1.88E-06 | 0.0487 | 0.0279 | 8.16E-02 | 0.0030 | 21.77 | TRUE | 1.20E-05 |
|  |  | rs3949865 | C | G | -0.0115 | 0.0026 | 9.49E-06 | 0.0423 | 0.0266 | 1.11E-01 | 0.0027 | 19.56 | TRUE | 3.22E-05 |
|  |  | rs4269521 | A | G | 0.0168 | 0.0034 | 1.03E-06 | 0.0310 | 0.0344 | 3.68E-01 | 0.0034 | 24.41 | TRUE | 1.88E-06 |
|  |  | rs4746017 | A | C | -0.0122 | 0.0027 | 5.10E-06 | -0.0367 | 0.0299 | 2.20E-01 | 0.0028 | 20.41 | TRUE | 1.69E-05 |
|  |  | rs6034318 | A | G | 0.1083 | 0.0236 | 4.52E-06 | -0.0672 | 0.0750 | 3.70E-01 | 0.0126 | 21.03 | TRUE | 6.56E-06 |
|  |  | rs6078500 | T | C | -0.0118 | 0.0026 | 4.47E-06 | 0.0085 | 0.0255 | 7.38E-01 | 0.0029 | 20.59 | TRUE | 8.75E-06 |
|  |  | rs6774965 | A | G | -0.0163 | 0.0035 | 3.16E-06 | 0.0070 | 0.0344 | 8.39E-01 | 0.0030 | 21.68 | TRUE | 4.62E-06 |
|  |  | rs6848070 | A | G | -0.0117 | 0.0027 | 9.91E-06 | 0.0515 | 0.0256 | 4.47E-02 | 0.0026 | 18.77 | TRUE | 6.04E-05 |
|  |  | rs7009776 | T | C | -0.0268 | 0.0060 | 7.12E-06 | 0.0606 | 0.0268 | 2.40E-02 | 0.0119 | 19.93 | TRUE | 1.75E-05 |
|  |  | rs7458704 | T | G | -0.0544 | 0.0109 | 5.60E-07 | 0.0604 | 0.0687 | 3.80E-01 | 0.0035 | 24.90 | TRUE | 1.45E-06 |
|  |  | rs7574257 | T | C | 0.0118 | 0.0026 | 7.76E-06 | 0.0201 | 0.0267 | 4.52E-01 | 0.0029 | 20.59 | TRUE | 1.14E-05 |
|  |  | rs8065435 | A | G | -0.0128 | 0.0028 | 5.03E-06 | 0.0138 | 0.0262 | 5.98E-01 | 0.0029 | 20.89 | TRUE | 8.52E-06 |
|  |  | rs9365106 | A | G | -0.0117 | 0.0026 | 8.06E-06 | 0.0183 | 0.0260 | 4.82E-01 | 0.0028 | 20.24 | TRUE | 1.32E-05 |
| M01564 | citrate | rs10043015 | T | C | 0.0082 | 0.0018 | 5.58E-06 | 0.0077 | 0.0283 | 7.86E-01 | 0.0028 | 20.75 | TRUE | 7.83E-06 |
|  |  | rs10787308 | A | G | -0.0090 | 0.0018 | 6.04E-07 | -0.0085 | 0.0303 | 7.80E-01 | 0.0034 | 24.99 | TRUE | 9.20E-07 |
|  |  | rs10992722 | A | C | 0.0326 | 0.0069 | 2.44E-06 | -0.0341 | 0.0759 | 6.53E-01 | 0.0030 | 22.32 | TRUE | 3.99E-06 |
|  |  | rs11120725 | A | G | -0.0181 | 0.0039 | 4.61E-06 | -0.0454 | 0.0257 | 7.71E-02 | 0.0121 | 21.51 | TRUE | 6.90E-06 |
|  |  | rs1125471 | A | C | -0.0085 | 0.0018 | 4.47E-06 | 0.0170 | 0.0267 | 5.24E-01 | 0.0030 | 22.29 | TRUE | 4.58E-06 |
|  |  | rs12121428 | T | C | -0.0197 | 0.0044 | 6.09E-06 | 0.0286 | 0.0548 | 6.01E-01 | 0.0027 | 20.04 | TRUE | 1.32E-05 |
|  |  | rs12625828 | T | G | 0.0086 | 0.0018 | 2.06E-06 | 0.0007 | 0.0262 | 9.79E-01 | 0.0031 | 22.82 | TRUE | 2.31E-06 |
|  |  | rs12803308 | A | T | -0.0081 | 0.0018 | 5.97E-06 | -0.0096 | 0.0268 | 7.21E-01 | 0.0027 | 20.24 | TRUE | 1.07E-05 |
|  |  | rs13130021 | T | C | -0.0089 | 0.0018 | 1.41E-06 | -0.0130 | 0.0262 | 6.21E-01 | 0.0033 | 24.44 | TRUE | 1.42E-06 |
|  |  | rs1380634 | A | G | -0.0083 | 0.0018 | 5.17E-06 | 0.0011 | 0.0348 | 9.76E-01 | 0.0029 | 21.26 | TRUE | 5.15E-06 |
|  |  | rs13966 | T | C | 0.0082 | 0.0019 | 9.99E-06 | -0.0226 | 0.0261 | 3.88E-01 | 0.0025 | 18.62 | TRUE | 3.33E-05 |
|  |  | rs1404373 | T | C | -0.0084 | 0.0018 | 2.92E-06 | -0.0203 | 0.0277 | 4.63E-01 | 0.0029 | 21.77 | TRUE | 6.34E-06 |
|  |  | rs1509123 | T | C | -0.0248 | 0.0043 | 5.20E-09 | -0.0157 | 0.0750 | 8.34E-01 | 0.0045 | 33.25 | TRUE | 1.38E-08 |
|  |  | rs1530219 | A | G | -0.0078 | 0.0018 | 9.94E-06 | 0.0087 | 0.0256 | 7.35E-01 | 0.0025 | 18.77 | TRUE | 2.23E-05 |
|  |  | rs170149 | A | G | 0.0256 | 0.0035 | 1.42E-13 | 0.0508 | 0.0661 | 4.42E-01 | 0.0072 | 53.48 | TRUE | 1.04E-12 |
|  |  | rs17034684 | T | G | 0.0081 | 0.0018 | 7.90E-06 | 0.0256 | 0.0264 | 3.34E-01 | 0.0027 | 20.24 | TRUE | 1.58E-05 |
|  |  | rs2143327 | T | C | 0.0392 | 0.0085 | 4.28E-06 | 0.1126 | 0.1478 | 4.46E-01 | 0.0029 | 21.26 | TRUE | 8.33E-06 |
|  |  | rs2236299 | A | G | 0.0087 | 0.0018 | 1.69E-06 | 0.0191 | 0.0331 | 5.62E-01 | 0.0032 | 23.35 | TRUE | 2.59E-06 |
|  |  | rs2239337 | C | G | 0.0230 | 0.0051 | 7.51E-06 | 0.0464 | 0.0575 | 4.20E-01 | 0.0028 | 20.33 | TRUE | 1.37E-05 |
|  |  | rs2370136 | T | C | -0.0086 | 0.0018 | 1.25E-06 | 0.0166 | 0.0263 | 5.28E-01 | 0.0031 | 22.82 | TRUE | 3.50E-06 |
|  |  | rs2534835 | T | G | -0.0083 | 0.0018 | 6.23E-06 | -0.0371 | 0.0316 | 2.41E-01 | 0.0029 | 21.26 | TRUE | 1.09E-05 |
|  |  | rs3193970 | T | C | 0.0081 | 0.0018 | 6.07E-06 | 0.0134 | 0.0261 | 6.07E-01 | 0.0027 | 20.24 | TRUE | 1.18E-05 |
|  |  | rs4235093 | A | G | -0.0083 | 0.0018 | 5.04E-06 | 0.0346 | 0.0285 | 2.24E-01 | 0.0029 | 21.26 | TRUE | 1.12E-05 |
|  |  | rs4589188 | A | G | 0.0122 | 0.0026 | 3.34E-06 | -0.0116 | 0.0307 | 7.06E-01 | 0.0030 | 22.01 | TRUE | 4.43E-06 |
|  |  | rs4779800 | A | G | -0.0122 | 0.0027 | 6.06E-06 | -0.0573 | 0.0373 | 1.25E-01 | 0.0028 | 20.41 | TRUE | 2.08E-05 |
|  |  | rs4999864 | C | G | 0.0179 | 0.0035 | 4.77E-07 | 0.1307 | 0.0575 | 2.30E-02 | 0.0035 | 26.15 | TRUE | 2.13E-06 |
|  |  | rs588973 | A | G | 0.0090 | 0.0020 | 6.80E-06 | -0.0013 | 0.0269 | 9.63E-01 | 0.0036 | 20.24 | TRUE | 8.31E-06 |
|  |  | rs6077792 | T | G | -0.0085 | 0.0018 | 2.82E-06 | -0.0313 | 0.0280 | 2.64E-01 | 0.0030 | 22.29 | TRUE | 6.31E-06 |
|  |  | rs6507399 | A | G | -0.0101 | 0.0018 | 1.70E-08 | -0.0044 | 0.0268 | 8.69E-01 | 0.0043 | 31.48 | TRUE | 3.24E-08 |
|  |  | rs6678076 | A | G | -0.0252 | 0.0052 | 1.08E-06 | -0.0285 | 0.0493 | 5.63E-01 | 0.0032 | 23.48 | TRUE | 2.43E-06 |
|  |  | rs7105815 | A | G | 0.0091 | 0.0018 | 4.93E-07 | 0.0263 | 0.0282 | 3.50E-01 | 0.0035 | 25.55 | TRUE | 1.11E-06 |
|  |  | rs7584253 | A | C | 0.0083 | 0.0018 | 5.04E-06 | -0.0010 | 0.0283 | 9.71E-01 | 0.0029 | 21.26 | TRUE | 5.17E-06 |
|  |  | rs7710027 | T | C | 0.0081 | 0.0018 | 9.16E-06 | -0.0073 | 0.0275 | 7.90E-01 | 0.0027 | 20.24 | TRUE | 1.01E-05 |
|  |  | rs7851531 | A | G | -0.0090 | 0.0018 | 6.47E-07 | 0.0312 | 0.0271 | 2.49E-01 | 0.0034 | 24.99 | TRUE | 1.71E-06 |
|  |  | rs7919076 | T | C | -0.0081 | 0.0018 | 7.79E-06 | -0.0392 | 0.0295 | 1.85E-01 | 0.0027 | 20.24 | TRUE | 1.98E-05 |
|  |  | rs8017368 | A | C | -0.0083 | 0.0018 | 3.85E-06 | 0.0081 | 0.0278 | 7.72E-01 | 0.0029 | 21.26 | TRUE | 6.12E-06 |
|  |  | rs807670 | A | C | 0.0110 | 0.0018 | 6.84E-10 | 0.0195 | 0.0259 | 4.53E-01 | 0.0050 | 37.34 | TRUE | 2.86E-09 |
|  |  | rs835154 | A | G | -0.0114 | 0.0018 | 1.15E-10 | -0.0471 | 0.0260 | 6.99E-02 | 0.0054 | 40.10 | TRUE | 1.86E-09 |
|  |  | rs889128 | T | G | 0.0082 | 0.0018 | 6.75E-06 | 0.0473 | 0.0261 | 6.99E-02 | 0.0028 | 20.75 | TRUE | 2.11E-05 |
|  |  | rs9327706 | A | C | 0.0081 | 0.0018 | 5.61E-06 | 0.0073 | 0.0258 | 7.76E-01 | 0.0027 | 20.24 | TRUE | 1.02E-05 |
|  |  | rs9372398 | T | C | 0.0081 | 0.0018 | 6.74E-06 | 0.0387 | 0.0259 | 1.35E-01 | 0.0027 | 20.24 | TRUE | 2.21E-05 |
|  |  | rs9389071 | A | G | -0.0120 | 0.0026 | 5.64E-06 | -0.0084 | 0.0382 | 8.27E-01 | 0.0029 | 21.30 | TRUE | 5.72E-06 |
|  |  | rs940697 | A | G | 0.0082 | 0.0018 | 5.71E-06 | 0.0002 | 0.0302 | 9.96E-01 | 0.0028 | 20.75 | TRUE | 6.57E-06 |
| M16822 | X-04499--3,4-dihydroxybutyrate | rs1009184 | T | G | -0.0123 | 0.0025 | 6.00E-07 | -0.0233 | 0.0257 | 3.65E-01 | 0.0037 | 24.20 | TRUE | 2.00E-06 |
|  |  | rs1030420 | T | C | -0.0221 | 0.0032 | 1.02E-11 | -0.0164 | 0.0345 | 6.34E-01 | 0.0072 | 47.68 | TRUE | 1.26E-11 |
|  |  | rs11856353 | T | C | -0.0162 | 0.0035 | 2.87E-06 | 0.0061 | 0.0378 | 8.72E-01 | 0.0033 | 21.42 | TRUE | 5.03E-06 |
|  |  | rs13332924 | A | C | 0.0132 | 0.0026 | 4.76E-07 | 0.0207 | 0.0285 | 4.68E-01 | 0.0039 | 25.77 | TRUE | 8.13E-07 |
|  |  | rs1525549 | A | G | 0.0166 | 0.0034 | 1.11E-06 | 0.0030 | 0.0389 | 9.38E-01 | 0.0036 | 23.83 | TRUE | 1.40E-06 |
|  |  | rs2333040 | A | G | 0.0115 | 0.0025 | 4.16E-06 | 0.0273 | 0.0262 | 2.99E-01 | 0.0032 | 21.15 | TRUE | 9.93E-06 |
|  |  | rs2372342 | A | G | 0.0217 | 0.0049 | 9.44E-06 | -0.0772 | 0.0642 | 2.29E-01 | 0.0030 | 19.61 | TRUE | 2.36E-05 |
|  |  | rs2632020 | T | C | 0.0109 | 0.0025 | 9.83E-06 | 0.0414 | 0.0257 | 1.07E-01 | 0.0029 | 19.00 | TRUE | 4.04E-05 |
|  |  | rs4273762 | T | C | -0.0182 | 0.0041 | 8.75E-06 | -0.0972 | 0.0542 | 7.32E-02 | 0.0030 | 19.70 | TRUE | 3.19E-05 |
|  |  | rs4325745 | T | C | -0.0140 | 0.0030 | 3.06E-06 | -0.0101 | 0.0259 | 6.97E-01 | 0.0044 | 21.77 | TRUE | 4.51E-06 |
|  |  | rs504192 | A | G | -0.0151 | 0.0034 | 7.68E-06 | 0.0204 | 0.0359 | 5.71E-01 | 0.0030 | 19.72 | TRUE | 1.53E-05 |
|  |  | rs504899 | T | C | -0.0232 | 0.0048 | 1.42E-06 | -0.0302 | 0.0570 | 5.96E-01 | 0.0036 | 23.35 | TRUE | 2.39E-06 |
|  |  | rs6024831 | A | G | -0.0189 | 0.0042 | 7.09E-06 | 0.0241 | 0.0387 | 5.34E-01 | 0.0031 | 20.24 | TRUE | 1.21E-05 |
|  |  | rs6748268 | T | C | 0.0112 | 0.0025 | 8.73E-06 | -0.0168 | 0.0268 | 5.32E-01 | 0.0031 | 20.06 | TRUE | 1.33E-05 |
|  |  | rs6959584 | T | C | 0.0173 | 0.0035 | 6.10E-07 | 0.0367 | 0.0312 | 2.38E-01 | 0.0037 | 24.42 | TRUE | 2.14E-06 |
|  |  | rs7699551 | T | C | -0.0118 | 0.0025 | 3.31E-06 | -0.0491 | 0.0268 | 6.68E-02 | 0.0034 | 22.27 | TRUE | 9.33E-06 |
|  |  | rs8112502 | A | G | -0.0212 | 0.0041 | 2.64E-07 | -0.0442 | 0.0417 | 2.89E-01 | 0.0041 | 26.73 | TRUE | 6.34E-07 |
|  |  | rs9393768 | A | G | 0.0114 | 0.0025 | 6.24E-06 | -0.0163 | 0.0280 | 5.59E-01 | 0.0032 | 20.79 | TRUE | 9.02E-06 |
|  |  | rs9525877 | A | G | -0.0118 | 0.0026 | 6.50E-06 | -0.0314 | 0.0299 | 2.94E-01 | 0.0031 | 20.59 | TRUE | 1.32E-05 |
|  |  | rs9604751 | A | G | 0.0180 | 0.0038 | 2.04E-06 | 0.0370 | 0.0305 | 2.26E-01 | 0.0034 | 22.43 | TRUE | 5.86E-06 |
| M19415 | X-06351 | rs12936996 | A | G | -0.0192 | 0.0038 | 5.86E-07 | -0.0067 | 0.0332 | 8.39E-01 | 0.0057 | 25.52 | TRUE | 5.90E-07 |
|  |  | rs17422462 | T | G | -0.0218 | 0.0048 | 4.62E-06 | -0.0263 | 0.0357 | 4.61E-01 | 0.0046 | 20.62 | TRUE | 9.45E-06 |
|  |  | rs2964431 | A | C | 0.0167 | 0.0037 | 7.85E-06 | 0.0334 | 0.0260 | 2.00E-01 | 0.0045 | 20.36 | TRUE | 1.42E-05 |
|  |  | rs3130663 | A | G | -0.1184 | 0.0247 | 1.55E-06 | -0.1534 | 0.1109 | 1.67E-01 | 0.0262 | 22.92 | TRUE | 2.64E-06 |
|  |  | rs4347230 | A | G | -0.0169 | 0.0037 | 6.04E-06 | -0.0273 | 0.0265 | 3.03E-01 | 0.0047 | 20.85 | TRUE | 9.74E-06 |
|  |  | rs7295482 | A | G | 0.0304 | 0.0064 | 1.77E-06 | 0.0277 | 0.0443 | 5.32E-01 | 0.0050 | 22.55 | TRUE | 3.35E-06 |
| M27728 | glycerol 2-phosphate | rs10117043 | T | G | 0.0161 | 0.0036 | 6.13E-06 | 0.0454 | 0.0337 | 1.78E-01 | 0.0036 | 19.99 | TRUE | 1.95E-05 |
|  |  | rs10953489 | T | C | -0.0157 | 0.0036 | 9.69E-06 | 0.0219 | 0.0323 | 4.98E-01 | 0.0034 | 19.01 | TRUE | 2.21E-05 |
|  |  | rs1105769 | A | C | -0.0135 | 0.0027 | 7.19E-07 | 0.0449 | 0.0263 | 8.80E-02 | 0.0045 | 24.99 | TRUE | 2.04E-06 |
|  |  | rs11078249 | T | C | -0.0125 | 0.0027 | 3.67E-06 | 0.0368 | 0.0262 | 1.60E-01 | 0.0038 | 21.43 | TRUE | 9.89E-06 |
|  |  | rs1234223 | C | G | 0.0211 | 0.0045 | 3.02E-06 | -0.0453 | 0.0438 | 3.01E-01 | 0.0039 | 21.98 | TRUE | 6.08E-06 |
|  |  | rs1317833 | A | G | -0.0125 | 0.0027 | 4.38E-06 | 0.0041 | 0.0259 | 8.73E-01 | 0.0038 | 21.43 | TRUE | 4.83E-06 |
|  |  | rs1401635 | C | G | -0.0125 | 0.0027 | 4.91E-06 | 0.0233 | 0.0271 | 3.90E-01 | 0.0038 | 21.43 | TRUE | 7.25E-06 |
|  |  | rs17359373 | T | C | -0.0323 | 0.0072 | 7.85E-06 | 0.0274 | 0.0303 | 3.66E-01 | 0.0161 | 20.09 | TRUE | 1.02E-05 |
|  |  | rs210331 | A | G | -0.0179 | 0.0035 | 3.53E-07 | -0.0008 | 0.0308 | 9.80E-01 | 0.0047 | 26.15 | TRUE | 4.01E-07 |
|  |  | rs2114588 | A | G | -0.0122 | 0.0027 | 6.99E-06 | 0.0263 | 0.0284 | 3.54E-01 | 0.0037 | 20.41 | TRUE | 1.25E-05 |
|  |  | rs2235396 | A | G | -0.0120 | 0.0027 | 9.66E-06 | -0.0059 | 0.0268 | 8.26E-01 | 0.0035 | 19.75 | TRUE | 1.18E-05 |
|  |  | rs2253834 | T | C | -0.0125 | 0.0027 | 4.55E-06 | 0.0353 | 0.0260 | 1.75E-01 | 0.0038 | 21.43 | TRUE | 9.63E-06 |
|  |  | rs2422239 | A | G | -0.0118 | 0.0027 | 9.52E-06 | 0.0320 | 0.0256 | 2.11E-01 | 0.0034 | 19.09 | TRUE | 2.90E-05 |
|  |  | rs2982376 | T | C | -0.0132 | 0.0028 | 2.93E-06 | 0.0356 | 0.0258 | 1.67E-01 | 0.0040 | 22.22 | TRUE | 6.60E-06 |
|  |  | rs4236964 | T | C | -0.0123 | 0.0027 | 5.74E-06 | 0.0008 | 0.0258 | 9.77E-01 | 0.0037 | 20.75 | TRUE | 6.35E-06 |
|  |  | rs4860118 | T | C | 0.0716 | 0.0151 | 2.04E-06 | -0.0726 | 0.0771 | 3.46E-01 | 0.0180 | 22.45 | TRUE | 3.11E-06 |
|  |  | rs4901357 | A | G | -0.0134 | 0.0028 | 1.25E-06 | 0.0162 | 0.0270 | 5.49E-01 | 0.0041 | 22.89 | TRUE | 2.97E-06 |
|  |  | rs498494 | C | G | 0.0258 | 0.0056 | 4.27E-06 | -0.0765 | 0.0336 | 2.26E-02 | 0.0038 | 21.22 | TRUE | 1.79E-05 |
|  |  | rs552279 | A | G | -0.0129 | 0.0028 | 4.90E-06 | -0.0008 | 0.0263 | 9.76E-01 | 0.0038 | 21.22 | TRUE | 4.98E-06 |
|  |  | rs6699475 | A | G | -0.0152 | 0.0034 | 8.91E-06 | 0.0450 | 0.0263 | 8.78E-02 | 0.0036 | 19.98 | TRUE | 2.39E-05 |
|  |  | rs7095787 | A | T | 0.0185 | 0.0035 | 1.58E-07 | 0.0436 | 0.0319 | 1.72E-01 | 0.0050 | 27.93 | TRUE | 3.90E-07 |
|  |  | rs7121554 | T | C | -0.0160 | 0.0035 | 5.21E-06 | 0.0261 | 0.0293 | 3.74E-01 | 0.0037 | 20.89 | TRUE | 9.65E-06 |
|  |  | rs7275210 | A | T | 0.0120 | 0.0027 | 9.31E-06 | 0.0048 | 0.0265 | 8.55E-01 | 0.0035 | 19.75 | TRUE | 1.16E-05 |
|  |  | rs734567 | T | C | 0.0134 | 0.0027 | 7.83E-07 | 0.0161 | 0.0260 | 5.35E-01 | 0.0044 | 24.62 | TRUE | 1.26E-06 |
|  |  | rs7428286 | A | G | -0.0561 | 0.0121 | 3.88E-06 | 0.3013 | 0.2072 | 1.46E-01 | 0.0038 | 21.49 | TRUE | 9.86E-06 |
|  |  | rs757704 | A | G | 0.0138 | 0.0027 | 2.22E-07 | 0.0070 | 0.0257 | 7.86E-01 | 0.0047 | 26.11 | TRUE | 4.78E-07 |
|  |  | rs7929077 | A | G | 0.0121 | 0.0027 | 6.63E-06 | -0.0187 | 0.0256 | 4.63E-01 | 0.0036 | 20.08 | TRUE | 1.33E-05 |
|  |  | rs908690 | T | C | -0.0161 | 0.0036 | 7.83E-06 | -0.0420 | 0.0367 | 2.52E-01 | 0.0036 | 19.99 | TRUE | 1.74E-05 |
|  |  | rs9471691 | T | C | 0.0139 | 0.0027 | 2.77E-07 | -0.0035 | 0.0260 | 8.94E-01 | 0.0047 | 26.49 | TRUE | 3.61E-07 |
|  |  | rs9608300 | C | G | -0.1370 | 0.0281 | 1.06E-06 | 0.1304 | 0.0720 | 7.02E-02 | 0.0190 | 23.73 | TRUE | 2.08E-06 |
| M32418 | myristoleate(14:1n5) | rs1009343 | A | C | 0.1003 | 0.0214 | 2.69E-06 | 0.0633 | 0.0462 | 1.71E-01 | 0.0123 | 21.94 | TRUE | 4.88E-06 |
|  |  | rs10098688 | A | C | 0.0267 | 0.0060 | 7.30E-06 | 0.0098 | 0.0255 | 7.01E-01 | 0.0111 | 19.78 | TRUE | 1.07E-05 |
|  |  | rs11728793 | T | C | -0.0210 | 0.0042 | 7.15E-07 | -0.0363 | 0.0276 | 1.89E-01 | 0.0034 | 24.99 | TRUE | 1.91E-06 |
|  |  | rs12539054 | T | C | -0.0175 | 0.0040 | 9.44E-06 | 0.0265 | 0.0342 | 4.38E-01 | 0.0026 | 19.14 | TRUE | 2.44E-05 |
|  |  | rs1508782 | A | C | 0.0232 | 0.0049 | 2.09E-06 | -0.0179 | 0.0381 | 6.38E-01 | 0.0030 | 22.41 | TRUE | 3.86E-06 |
|  |  | rs35350 | A | G | 0.0622 | 0.0132 | 2.33E-06 | 0.1668 | 0.0910 | 6.68E-02 | 0.0030 | 22.20 | TRUE | 1.06E-05 |
|  |  | rs4129516 | T | C | -0.0182 | 0.0040 | 5.51E-06 | -0.0312 | 0.0284 | 2.72E-01 | 0.0028 | 20.70 | TRUE | 1.37E-05 |
|  |  | rs4612338 | A | G | -0.0367 | 0.0078 | 2.44E-06 | 0.0140 | 0.0369 | 7.04E-01 | 0.0124 | 22.11 | TRUE | 3.22E-06 |
|  |  | rs4755163 | A | G | 0.0152 | 0.0033 | 3.51E-06 | -0.0085 | 0.0256 | 7.39E-01 | 0.0029 | 21.21 | TRUE | 6.45E-06 |
|  |  | rs603424 | A | G | -0.0368 | 0.0046 | 7.16E-16 | -0.0728 | 0.0402 | 6.96E-02 | 0.0086 | 63.98 | TRUE | 1.95E-14 |
|  |  | rs6075489 | A | G | 0.0185 | 0.0041 | 5.76E-06 | 0.0385 | 0.0294 | 1.90E-01 | 0.0028 | 20.35 | TRUE | 1.86E-05 |
|  |  | rs7100861 | T | C | -0.0268 | 0.0058 | 3.53E-06 | 0.0274 | 0.0261 | 2.94E-01 | 0.0119 | 21.33 | TRUE | 6.00E-06 |
|  |  | rs8088457 | A | G | 0.0496 | 0.0103 | 1.59E-06 | 0.0105 | 0.0791 | 8.94E-01 | 0.0031 | 23.18 | TRUE | 2.07E-06 |
|  |  | rs9691574 | T | C | 0.0300 | 0.0067 | 8.79E-06 | -0.0015 | 0.0415 | 9.70E-01 | 0.0027 | 20.04 | TRUE | 9.61E-06 |
|  |  | rs9888349 | T | C | -0.0192 | 0.0040 | 2.02E-06 | -0.0511 | 0.0296 | 8.40E-02 | 0.0031 | 23.03 | TRUE | 6.57E-06 |
| M32425 | dehydroisoandrosterone sulfate (DHEA-S) | rs10236826 | T | C | -0.0585 | 0.0125 | 2.68E-06 | 0.0912 | 0.0466 | 5.06E-02 | 0.0030 | 21.90 | TRUE | 1.32E-05 |
|  |  | rs10764712 | T | G | 0.0219 | 0.0049 | 9.62E-06 | -0.0320 | 0.0256 | 2.11E-01 | 0.0027 | 19.97 | TRUE | 2.16E-05 |
|  |  | rs11761528 | T | C | -0.0675 | 0.0090 | 6.70E-14 | 0.0248 | 0.0364 | 4.95E-01 | 0.0076 | 56.23 | TRUE | 2.48E-13 |
|  |  | rs12121588 | A | G | -0.0289 | 0.0058 | 5.43E-07 | 0.0327 | 0.0287 | 2.56E-01 | 0.0034 | 24.82 | TRUE | 1.83E-06 |
|  |  | rs12601482 | T | C | -0.0342 | 0.0075 | 4.43E-06 | -0.0469 | 0.0372 | 2.08E-01 | 0.0028 | 20.79 | TRUE | 1.45E-05 |
|  |  | rs13072891 | A | G | 0.0243 | 0.0049 | 7.95E-07 | -0.0043 | 0.0262 | 8.69E-01 | 0.0033 | 24.59 | TRUE | 1.04E-06 |
|  |  | rs13222543 | T | C | -0.1015 | 0.0199 | 3.49E-07 | -0.0894 | 0.0756 | 2.37E-01 | 0.0035 | 26.01 | TRUE | 1.06E-06 |
|  |  | rs1390947 | A | C | -0.0242 | 0.0049 | 6.96E-07 | -0.0304 | 0.0258 | 2.39E-01 | 0.0033 | 24.38 | TRUE | 2.34E-06 |
|  |  | rs17223039 | A | G | 0.0370 | 0.0083 | 8.92E-06 | -0.0026 | 0.0405 | 9.48E-01 | 0.0027 | 19.87 | TRUE | 1.07E-05 |
|  |  | rs1834260 | T | C | 0.0231 | 0.0052 | 7.30E-06 | 0.0022 | 0.0295 | 9.41E-01 | 0.0027 | 19.73 | TRUE | 1.16E-05 |
|  |  | rs2048428 | T | C | -0.0361 | 0.0078 | 3.55E-06 | 0.0111 | 0.0408 | 7.86E-01 | 0.0029 | 21.41 | TRUE | 5.58E-06 |
|  |  | rs296396 | T | C | -0.0462 | 0.0065 | 1.17E-12 | 0.0439 | 0.0361 | 2.24E-01 | 0.0068 | 50.51 | TRUE | 6.98E-12 |
|  |  | rs356296 | A | G | -0.0882 | 0.0194 | 5.57E-06 | -0.0535 | 0.1771 | 7.63E-01 | 0.0028 | 20.66 | TRUE | 8.33E-06 |
|  |  | rs3802037 | A | G | -0.0220 | 0.0050 | 9.93E-06 | -0.0182 | 0.0276 | 5.09E-01 | 0.0026 | 19.35 | TRUE | 2.03E-05 |
|  |  | rs628137 | A | C | -0.0221 | 0.0049 | 6.69E-06 | 0.0039 | 0.0260 | 8.81E-01 | 0.0028 | 20.34 | TRUE | 8.91E-06 |
|  |  | rs6900835 | T | C | -0.0273 | 0.0057 | 2.02E-06 | 0.0134 | 0.0312 | 6.68E-01 | 0.0031 | 22.93 | TRUE | 2.88E-06 |
|  |  | rs794614 | A | G | 0.0292 | 0.0058 | 5.44E-07 | -0.0174 | 0.0349 | 6.18E-01 | 0.0034 | 25.34 | TRUE | 9.04E-07 |
|  |  | rs878983 | T | C | -0.0261 | 0.0059 | 9.50E-06 | 0.0072 | 0.0275 | 7.92E-01 | 0.0027 | 19.56 | TRUE | 1.42E-05 |
|  |  | rs9316766 | A | G | -0.0270 | 0.0058 | 2.82E-06 | 0.0311 | 0.0288 | 2.81E-01 | 0.0029 | 21.66 | TRUE | 8.38E-06 |
|  |  | rs932412 | A | C | -0.0222 | 0.0049 | 5.62E-06 | 0.0188 | 0.0258 | 4.68E-01 | 0.0028 | 20.52 | TRUE | 1.18E-05 |
|  |  | rs9972424 | A | G | -0.0228 | 0.0050 | 4.63E-06 | 0.0279 | 0.0265 | 2.92E-01 | 0.0028 | 20.79 | TRUE | 1.27E-05 |
| M32754 | X-11437 | rs10517818 | T | C | -0.0501 | 0.0111 | 6.62E-06 | -0.0546 | 0.0365 | 1.34E-01 | 0.0032 | 20.37 | TRUE | 1.91E-05 |
|  |  | rs12435324 | C | G | -0.4219 | 0.0908 | 3.37E-06 | 0.0127 | 0.0761 | 8.67E-01 | 0.0155 | 21.56 | TRUE | 3.95E-06 |
|  |  | rs13073145 | C | G | -0.0970 | 0.0219 | 9.48E-06 | -0.0518 | 0.0538 | 3.36E-01 | 0.0031 | 19.61 | TRUE | 2.02E-05 |
|  |  | rs1405215 | A | G | 0.0623 | 0.0116 | 8.00E-08 | -0.0597 | 0.0339 | 7.76E-02 | 0.0045 | 28.84 | TRUE | 3.60E-07 |
|  |  | rs16926956 | T | G | -0.0520 | 0.0109 | 1.77E-06 | 0.0160 | 0.0311 | 6.06E-01 | 0.0036 | 22.75 | TRUE | 3.18E-06 |
|  |  | rs17040357 | A | G | 0.3091 | 0.0679 | 5.38E-06 | -0.0810 | 0.0440 | 6.56E-02 | 0.0149 | 20.69 | TRUE | 9.94E-06 |
|  |  | rs17825729 | C | G | -0.0711 | 0.0153 | 3.36E-06 | 0.0250 | 0.0554 | 6.52E-01 | 0.0034 | 21.59 | TRUE | 5.49E-06 |
|  |  | rs2460621 | T | C | 0.0795 | 0.0180 | 9.84E-06 | 0.0438 | 0.0433 | 3.12E-01 | 0.0030 | 19.50 | TRUE | 2.20E-05 |
|  |  | rs335810 | A | C | 0.3722 | 0.0827 | 6.80E-06 | -0.1810 | 0.1545 | 2.41E-01 | 0.0146 | 20.23 | TRUE | 1.04E-05 |
|  |  | rs4919957 | A | G | 0.1629 | 0.0358 | 5.40E-06 | -0.0180 | 0.0408 | 6.60E-01 | 0.0149 | 20.67 | TRUE | 6.74E-06 |
|  |  | rs555851 | A | G | -0.0363 | 0.0082 | 9.23E-06 | 0.0351 | 0.0257 | 1.71E-01 | 0.0031 | 19.59 | TRUE | 2.59E-05 |
|  |  | rs6415698 | A | C | 0.0548 | 0.0107 | 3.10E-07 | -0.0495 | 0.0313 | 1.13E-01 | 0.0041 | 26.22 | TRUE | 1.14E-06 |
|  |  | rs7321884 | T | C | 0.1488 | 0.0316 | 2.43E-06 | -0.0543 | 0.0807 | 5.01E-01 | 0.0035 | 22.17 | TRUE | 4.71E-06 |
|  |  | rs768328 | T | C | -0.0464 | 0.0099 | 3.09E-06 | -0.0008 | 0.0313 | 9.79E-01 | 0.0034 | 21.96 | TRUE | 3.48E-06 |
|  |  | rs9543952 | A | G | -0.0530 | 0.0117 | 5.80E-06 | 0.0691 | 0.0356 | 5.24E-02 | 0.0032 | 20.51 | TRUE | 2.29E-05 |
| M33132 | X-11787 | rs10206899 | T | C | 0.0399 | 0.0017 | 7.74E-123 | -0.0041 | 0.0335 | 9.02E-01 | 0.0696 | 550.72 | TRUE | 1.93E-116 |
|  |  | rs10476724 | T | C | 0.0078 | 0.0018 | 9.43E-06 | -0.0336 | 0.0416 | 4.20E-01 | 0.0025 | 18.77 | TRUE | 2.98E-05 |
|  |  | rs10920716 | A | G | -0.0178 | 0.0038 | 2.27E-06 | 0.0376 | 0.0313 | 2.30E-01 | 0.0123 | 21.92 | TRUE | 4.67E-06 |
|  |  | rs11073472 | A | G | -0.0092 | 0.0018 | 5.55E-07 | -0.0004 | 0.0356 | 9.91E-01 | 0.0035 | 26.12 | TRUE | 4.29E-07 |
|  |  | rs11187969 | A | G | 0.0095 | 0.0018 | 2.05E-07 | -0.0362 | 0.0474 | 4.45E-01 | 0.0038 | 27.85 | TRUE | 3.15E-07 |
|  |  | rs11813961 | A | G | -0.0119 | 0.0027 | 9.12E-06 | 0.0205 | 0.0440 | 6.41E-01 | 0.0026 | 19.42 | TRUE | 1.74E-05 |
|  |  | rs12902975 | T | C | -0.0115 | 0.0025 | 4.19E-06 | 0.0067 | 0.0339 | 8.43E-01 | 0.0029 | 21.15 | TRUE | 6.07E-06 |
|  |  | rs17044007 | T | G | -0.0140 | 0.0030 | 3.06E-06 | 0.0000 | 0.0606 | 1.00E+00 | 0.0039 | 21.77 | TRUE | 3.69E-06 |
|  |  | rs17110031 | T | C | 0.0350 | 0.0034 | 1.10E-24 | -0.0679 | 0.0894 | 4.48E-01 | 0.0142 | 105.94 | TRUE | 8.27E-24 |
|  |  | rs17168815 | T | G | -0.0084 | 0.0018 | 3.68E-06 | 0.0385 | 0.0322 | 2.32E-01 | 0.0029 | 21.77 | TRUE | 8.59E-06 |
|  |  | rs17423838 | A | G | 0.0086 | 0.0018 | 3.06E-06 | -0.0596 | 0.0329 | 6.98E-02 | 0.0031 | 22.82 | TRUE | 7.72E-06 |
|  |  | rs17490468 | A | G | -0.0173 | 0.0036 | 1.22E-06 | -0.0904 | 0.0615 | 1.42E-01 | 0.0031 | 23.09 | TRUE | 5.40E-06 |
|  |  | rs17595027 | T | C | -0.0080 | 0.0017 | 4.76E-06 | -0.0135 | 0.0420 | 7.48E-01 | 0.0030 | 22.14 | TRUE | 4.00E-06 |
|  |  | rs17718517 | T | C | 0.0090 | 0.0020 | 6.80E-06 | 0.0096 | 0.0317 | 7.62E-01 | 0.0036 | 20.24 | TRUE | 9.61E-06 |
|  |  | rs1927544 | T | C | 0.0078 | 0.0018 | 9.67E-06 | 0.0120 | 0.0293 | 6.82E-01 | 0.0025 | 18.77 | TRUE | 2.33E-05 |
|  |  | rs1934963 | T | C | 0.0293 | 0.0017 | 8.69E-65 | -0.0538 | 0.0328 | 1.01E-01 | 0.0388 | 296.97 | TRUE | 9.33E-63 |
|  |  | rs2009767 | T | C | 0.0092 | 0.0018 | 4.14E-07 | 0.0210 | 0.0385 | 5.85E-01 | 0.0035 | 26.12 | TRUE | 6.34E-07 |
|  |  | rs2071426 | T | C | 0.0084 | 0.0017 | 3.75E-07 | -0.0223 | 0.0286 | 4.35E-01 | 0.0033 | 24.41 | TRUE | 1.76E-06 |
|  |  | rs2160166 | T | C | 0.0077 | 0.0017 | 8.01E-06 | -0.0039 | 0.0284 | 8.91E-01 | 0.0028 | 20.51 | TRUE | 8.09E-06 |
|  |  | rs2924762 | T | C | 0.0086 | 0.0018 | 2.25E-06 | 0.0347 | 0.0450 | 4.41E-01 | 0.0031 | 22.82 | TRUE | 3.85E-06 |
|  |  | rs4521827 | A | T | 0.0085 | 0.0018 | 2.17E-06 | -0.0012 | 0.0401 | 9.77E-01 | 0.0030 | 22.29 | TRUE | 3.03E-06 |
|  |  | rs4900819 | T | C | -0.0097 | 0.0018 | 6.88E-08 | 0.0202 | 0.0356 | 5.70E-01 | 0.0039 | 29.03 | TRUE | 1.51E-07 |
|  |  | rs6507259 | T | C | -0.0078 | 0.0018 | 9.52E-06 | -0.0044 | 0.0406 | 9.14E-01 | 0.0025 | 18.77 | TRUE | 1.93E-05 |
|  |  | rs666276 | T | C | -0.0094 | 0.0018 | 2.19E-07 | 0.0693 | 0.0424 | 1.03E-01 | 0.0037 | 27.26 | TRUE | 7.91E-07 |
|  |  | rs6969691 | A | G | -0.0083 | 0.0018 | 5.29E-06 | 0.0660 | 0.0502 | 1.89E-01 | 0.0029 | 21.26 | TRUE | 1.20E-05 |
|  |  | rs739626 | A | C | 0.0130 | 0.0024 | 7.96E-08 | -0.0419 | 0.0331 | 2.06E-01 | 0.0040 | 29.33 | TRUE | 2.20E-07 |
|  |  | rs7794651 | T | C | 0.0089 | 0.0018 | 7.66E-07 | 0.0078 | 0.0372 | 8.33E-01 | 0.0033 | 24.44 | TRUE | 1.16E-06 |
|  |  | rs7992587 | T | C | -0.0083 | 0.0017 | 7.72E-07 | -0.0173 | 0.0283 | 5.41E-01 | 0.0032 | 23.83 | TRUE | 2.08E-06 |
|  |  | rs932748 | T | G | 0.0090 | 0.0020 | 6.80E-06 | -0.0642 | 0.0341 | 6.00E-02 | 0.0036 | 20.24 | TRUE | 2.32E-05 |
|  |  | rs9686531 | T | C | 0.0081 | 0.0018 | 7.74E-06 | 0.0523 | 0.0410 | 2.03E-01 | 0.0027 | 20.24 | TRUE | 1.92E-05 |
|  |  | rs994713 | T | C | -0.0204 | 0.0045 | 6.37E-06 | 0.0982 | 0.1148 | 3.92E-01 | 0.0028 | 20.55 | TRUE | 1.27E-05 |
| M33610 | X-12189 | rs10425480 | A | G | 0.5394 | 0.0954 | 1.59E-08 | 0.1811 | 0.1648 | 2.72E-01 | 0.2386 | 31.34 | TRUE | 9.11E-08 |
|  |  | rs10836708 | C | G | -0.1272 | 0.0280 | 5.53E-06 | -0.0342 | 0.0263 | 1.94E-01 | 0.1683 | 20.23 | TRUE | 1.33E-05 |
|  |  | rs11747400 | A | T | 0.2269 | 0.0461 | 8.78E-07 | 0.0477 | 0.0863 | 5.81E-01 | 0.0490 | 24.12 | TRUE | 1.22E-06 |
|  |  | rs12498013 | T | C | 0.3027 | 0.0618 | 9.79E-07 | -0.0592 | 0.1003 | 5.55E-01 | 0.1904 | 23.52 | TRUE | 2.78E-06 |
|  |  | rs1408509 | A | G | 0.0526 | 0.0110 | 1.78E-06 | 0.0417 | 0.0286 | 1.44E-01 | 0.0464 | 22.77 | TRUE | 2.82E-06 |
|  |  | rs1572704 | A | G | 0.3904 | 0.0764 | 3.29E-07 | 0.1794 | 0.0978 | 6.65E-02 | 0.2038 | 25.60 | TRUE | 1.21E-06 |
|  |  | rs1596834 | A | C | 0.1944 | 0.0435 | 7.87E-06 | -0.0017 | 0.0405 | 9.67E-01 | 0.1637 | 19.58 | TRUE | 1.64E-05 |
|  |  | rs16829299 | T | G | 0.3861 | 0.0870 | 9.12E-06 | -0.0505 | 0.1339 | 7.06E-01 | 0.1618 | 19.31 | TRUE | 1.91E-05 |
|  |  | rs17594255 | A | T | -0.0742 | 0.0156 | 2.10E-06 | -0.0080 | 0.0418 | 8.47E-01 | 0.0459 | 22.53 | TRUE | 2.55E-06 |
|  |  | rs2803893 | A | G | -0.0621 | 0.0138 | 7.09E-06 | -0.0137 | 0.0314 | 6.62E-01 | 0.0413 | 20.16 | TRUE | 8.79E-06 |
|  |  | rs321188 | A | G | -0.5360 | 0.1117 | 1.59E-06 | -0.0330 | 0.1125 | 7.69E-01 | 0.1842 | 22.57 | TRUE | 4.17E-06 |
|  |  | rs357118 | A | G | 0.1438 | 0.0308 | 3.03E-06 | -0.0054 | 0.0345 | 8.75E-01 | 0.1761 | 21.37 | TRUE | 7.18E-06 |
|  |  | rs4291236 | C | G | 0.2588 | 0.0570 | 5.54E-06 | -0.0241 | 0.0476 | 6.13E-01 | 0.1681 | 20.21 | TRUE | 1.26E-05 |
|  |  | rs4458182 | A | T | -0.0862 | 0.0189 | 5.30E-06 | -0.0699 | 0.0557 | 2.10E-01 | 0.0424 | 20.71 | TRUE | 7.64E-06 |
|  |  | rs5022652 | A | G | 0.0649 | 0.0140 | 3.84E-06 | 0.0627 | 0.0304 | 3.95E-02 | 0.0437 | 21.40 | TRUE | 6.20E-06 |
|  |  | rs601208 | T | C | 0.0518 | 0.0111 | 3.04E-06 | -0.0122 | 0.0291 | 6.74E-01 | 0.0443 | 21.69 | TRUE | 4.06E-06 |
|  |  | rs6679176 | T | C | -0.3414 | 0.0696 | 9.20E-07 | -0.0705 | 0.0316 | 2.60E-02 | 0.1909 | 23.59 | TRUE | 3.07E-06 |
|  |  | rs6803591 | T | C | 0.3527 | 0.0781 | 6.25E-06 | 0.0477 | 0.0819 | 5.60E-01 | 0.1666 | 19.99 | TRUE | 1.41E-05 |
|  |  | rs7105058 | A | G | 0.0532 | 0.0115 | 3.36E-06 | 0.0060 | 0.0256 | 8.16E-01 | 0.0436 | 21.31 | TRUE | 4.75E-06 |
|  |  | rs7147680 | A | G | 0.5941 | 0.1197 | 6.94E-07 | -0.0521 | 0.1946 | 7.89E-01 | 0.1945 | 24.15 | TRUE | 2.03E-06 |
|  |  | rs7185611 | T | C | -0.4842 | 0.0987 | 9.41E-07 | -0.0886 | 0.1265 | 4.84E-01 | 0.1909 | 23.59 | TRUE | 2.71E-06 |
|  |  | rs7222177 | T | C | 0.5557 | 0.1220 | 5.20E-06 | 0.0710 | 0.1261 | 5.74E-01 | 0.1690 | 20.34 | TRUE | 1.19E-05 |
|  |  | rs7870108 | T | G | 0.6206 | 0.1180 | 1.46E-07 | 0.0224 | 0.0964 | 8.17E-01 | 0.2133 | 27.12 | TRUE | 5.36E-07 |
|  |  | rs8003714 | T | G | 0.4616 | 0.0995 | 3.46E-06 | 0.1475 | 0.1292 | 2.54E-01 | 0.1742 | 21.10 | TRUE | 8.78E-06 |
|  |  | rs8036131 | T | G | 0.0608 | 0.0130 | 3.15E-06 | 0.0033 | 0.0277 | 9.05E-01 | 0.0445 | 21.78 | TRUE | 3.67E-06 |
|  |  | rs913080 | T | C | 0.1166 | 0.0245 | 1.97E-06 | 0.0077 | 0.0281 | 7.86E-01 | 0.1817 | 22.21 | TRUE | 4.93E-06 |
|  |  | rs9833839 | T | C | 0.2858 | 0.0620 | 3.96E-06 | -0.3053 | 0.2796 | 2.75E-01 | 0.0433 | 21.16 | TRUE | 5.94E-06 |
| M33969 | stearidonate(18:4n3) | rs11236516 | T | C | 0.0283 | 0.0058 | 1.24E-06 | -0.0068 | 0.0413 | 8.70E-01 | 0.0032 | 23.80 | TRUE | 1.55E-06 |
|  |  | rs12583004 | T | C | -0.0314 | 0.0071 | 9.90E-06 | -0.0664 | 0.0541 | 2.20E-01 | 0.0027 | 19.55 | TRUE | 2.62E-05 |
|  |  | rs174601 | T | C | -0.0336 | 0.0042 | 7.93E-16 | 0.0197 | 0.0259 | 4.47E-01 | 0.0087 | 63.98 | TRUE | 6.07E-15 |
|  |  | rs2265047 | A | G | 0.0335 | 0.0075 | 8.71E-06 | -0.0334 | 0.0273 | 2.22E-01 | 0.0112 | 19.93 | TRUE | 1.29E-05 |
|  |  | rs2427612 | T | C | -0.0286 | 0.0061 | 3.01E-06 | 0.0078 | 0.0265 | 7.67E-01 | 0.0030 | 21.98 | TRUE | 4.26E-06 |
|  |  | rs3750045 | T | C | 0.0266 | 0.0060 | 9.08E-06 | -0.0875 | 0.0513 | 8.76E-02 | 0.0027 | 19.65 | TRUE | 3.36E-05 |
|  |  | rs4529031 | T | C | -0.0841 | 0.0186 | 6.41E-06 | 0.0178 | 0.0405 | 6.61E-01 | 0.0115 | 20.42 | TRUE | 7.81E-06 |
|  |  | rs4811800 | A | G | -0.0212 | 0.0045 | 2.52E-06 | 0.0400 | 0.0257 | 1.20E-01 | 0.0030 | 22.19 | TRUE | 8.84E-06 |
|  |  | rs6473533 | T | G | -0.0217 | 0.0049 | 9.08E-06 | 0.0476 | 0.0288 | 9.84E-02 | 0.0027 | 19.61 | TRUE | 3.31E-05 |
|  |  | rs6986249 | T | C | -0.0177 | 0.0036 | 6.30E-07 | 0.0429 | 0.0255 | 9.30E-02 | 0.0033 | 24.17 | TRUE | 3.66E-06 |
|  |  | rs7142028 | A | C | -0.0161 | 0.0035 | 4.06E-06 | 0.0051 | 0.0266 | 8.47E-01 | 0.0029 | 21.15 | TRUE | 6.04E-06 |
| M34245 | X-12645 | rs10512731 | C | G | 0.0261 | 0.0054 | 1.15E-06 | -0.0220 | 0.0312 | 4.82E-01 | 0.0047 | 23.35 | TRUE | 2.41E-06 |
|  |  | rs10977991 | A | G | 0.0326 | 0.0073 | 8.88E-06 | 0.0228 | 0.0500 | 6.48E-01 | 0.0040 | 19.93 | TRUE | 1.19E-05 |
|  |  | rs11068695 | A | G | -0.0395 | 0.0083 | 2.06E-06 | -0.0105 | 0.0521 | 8.40E-01 | 0.0046 | 22.64 | TRUE | 2.60E-06 |
|  |  | rs12234425 | A | C | -0.0243 | 0.0055 | 9.33E-06 | 0.0092 | 0.0382 | 8.09E-01 | 0.0040 | 19.51 | TRUE | 1.31E-05 |
|  |  | rs12886782 | A | G | -0.0206 | 0.0046 | 8.11E-06 | -0.0130 | 0.0311 | 6.77E-01 | 0.0041 | 20.05 | TRUE | 1.10E-05 |
|  |  | rs1461941 | C | G | 0.0226 | 0.0045 | 5.01E-07 | -0.0164 | 0.0268 | 5.41E-01 | 0.0051 | 25.21 | TRUE | 8.94E-07 |
|  |  | rs1630427 | C | G | -0.0289 | 0.0064 | 7.48E-06 | -0.0250 | 0.0494 | 6.13E-01 | 0.0041 | 20.38 | TRUE | 9.69E-06 |
|  |  | rs168190 | T | C | 0.0465 | 0.0097 | 1.81E-06 | -0.0402 | 0.0259 | 1.22E-01 | 0.0283 | 22.92 | TRUE | 2.72E-06 |
|  |  | rs16852455 | T | G | -0.0164 | 0.0037 | 8.82E-06 | 0.0099 | 0.0256 | 6.99E-01 | 0.0040 | 19.64 | TRUE | 1.33E-05 |
|  |  | rs2070895 | A | G | 0.0250 | 0.0054 | 3.09E-06 | -0.0384 | 0.0293 | 1.90E-01 | 0.0043 | 21.42 | TRUE | 8.83E-06 |
|  |  | rs2912035 | T | G | 0.0218 | 0.0045 | 1.30E-06 | -0.0013 | 0.0260 | 9.59E-01 | 0.0048 | 23.46 | TRUE | 1.57E-06 |
|  |  | rs2980872 | A | G | -0.0180 | 0.0040 | 6.80E-06 | 0.0414 | 0.0260 | 1.12E-01 | 0.0049 | 20.24 | TRUE | 1.69E-05 |
|  |  | rs308589 | A | G | -0.0196 | 0.0044 | 9.20E-06 | 0.0160 | 0.0257 | 5.35E-01 | 0.0040 | 19.83 | TRUE | 1.36E-05 |
|  |  | rs311496 | T | C | -0.0338 | 0.0072 | 3.05E-06 | -0.0130 | 0.0293 | 6.58E-01 | 0.0045 | 22.03 | TRUE | 4.06E-06 |
|  |  | rs317279 | A | G | 0.0202 | 0.0045 | 7.62E-06 | -0.0327 | 0.0273 | 2.31E-01 | 0.0041 | 20.14 | TRUE | 1.58E-05 |
|  |  | rs6004798 | T | C | -0.0201 | 0.0045 | 7.14E-06 | 0.0318 | 0.0268 | 2.34E-01 | 0.0040 | 19.94 | TRUE | 1.73E-05 |
|  |  | rs7796052 | A | T | 0.0210 | 0.0046 | 4.84E-06 | 0.0293 | 0.0365 | 4.22E-01 | 0.0042 | 20.83 | TRUE | 9.05E-06 |
|  |  | rs807741 | T | G | -0.0805 | 0.0167 | 1.38E-06 | 0.0076 | 0.0455 | 8.68E-01 | 0.0286 | 23.18 | TRUE | 1.74E-06 |
|  |  | rs923673 | A | C | 0.0235 | 0.0046 | 2.98E-07 | -0.0493 | 0.0295 | 9.50E-02 | 0.0053 | 26.09 | TRUE | 1.07E-06 |
| M37097 | tryptophan betaine | rs10079835 | T | G | -0.1332 | 0.0258 | 2.33E-07 | -0.0299 | 0.1120 | 7.89E-01 | 0.0038 | 26.65 | TRUE | 3.90E-07 |
|  |  | rs12320124 | A | C | -0.2943 | 0.0664 | 9.38E-06 | 0.3858 | 0.2973 | 1.94E-01 | 0.0116 | 19.62 | TRUE | 1.53E-05 |
|  |  | rs13184973 | A | T | -0.0763 | 0.0138 | 3.29E-08 | 0.0107 | 0.0437 | 8.06E-01 | 0.0043 | 30.56 | TRUE | 5.37E-08 |
|  |  | rs2212465 | A | T | -0.0927 | 0.0207 | 7.30E-06 | 0.0065 | 0.0657 | 9.22E-01 | 0.0029 | 20.05 | TRUE | 9.87E-06 |
|  |  | rs2255908 | A | G | 0.0466 | 0.0088 | 1.04E-07 | -0.0078 | 0.0275 | 7.77E-01 | 0.0040 | 28.03 | TRUE | 1.96E-07 |
|  |  | rs2405522 | A | G | -0.1264 | 0.0113 | 4.49E-29 | -0.0327 | 0.0372 | 3.79E-01 | 0.0175 | 125.09 | TRUE | 8.66E-28 |
|  |  | rs3821142 | T | C | 0.0747 | 0.0157 | 2.09E-06 | 0.0262 | 0.0263 | 3.18E-01 | 0.0134 | 22.61 | TRUE | 3.05E-06 |
|  |  | rs3828671 | A | G | -0.1357 | 0.0257 | 1.24E-07 | -0.0654 | 0.0556 | 2.40E-01 | 0.0040 | 27.87 | TRUE | 4.08E-07 |
|  |  | rs4792110 | T | G | 0.1140 | 0.0252 | 6.31E-06 | 0.0548 | 0.0429 | 2.02E-01 | 0.0029 | 20.46 | TRUE | 1.68E-05 |
|  |  | rs4898658 | A | C | -0.0777 | 0.0164 | 2.05E-06 | -0.0638 | 0.0541 | 2.38E-01 | 0.0032 | 22.44 | TRUE | 5.94E-06 |
|  |  | rs719672 | A | C | 0.0917 | 0.0196 | 2.91E-06 | 0.0137 | 0.0301 | 6.49E-01 | 0.0129 | 21.86 | TRUE | 3.73E-06 |
|  |  | rs7582926 | T | C | -0.0437 | 0.0095 | 3.89E-06 | -0.0325 | 0.0295 | 2.71E-01 | 0.0030 | 21.15 | TRUE | 1.07E-05 |
|  |  | rs9651696 | T | C | -0.0756 | 0.0156 | 1.20E-06 | -0.0245 | 0.0256 | 3.39E-01 | 0.0139 | 23.46 | TRUE | 1.96E-06 |

**Supplemental table 4. Outliers identified by Radial MR.**

| **Metabolite ID** | **Metabolites** | **outliers** | **Q statistic** | ***p*-value** |
| --- | --- | --- | --- | --- |
| M00063 | cholesterol | rs1411983 | 7.2988 | 0.0069 |
| M01358 | stearate (18:0) | rs11940037 | 4.5896 | 0.0322 |
|  | stearate (18:0) | rs6453594 | 5.2174 | 0.0224 |
| M01564 | citrate | rs10514735 | 4.0083 | 0.0453 |
|  | citrate | rs868395 | 4.4829 | 0.0342 |
|  | citrate | rs910616 | 4.3370 | 0.0373 |
| M27728 | glycerol 2-phosphate | rs2041992 | 5.2449 | 0.0220 |
|  | glycerol 2-phosphate | rs8124907 | 6.5442 | 0.0105 |
| M32425 | dehydroisoandrosterone sulfate (DHEA-S) | rs10045690 | 6.7491 | 0.0094 |
| M33610 | X-12189 | rs11938671 | 6.7282 | 0.0095 |
|  |  | rs8082167 | 5.1611 | 0.0231 |

**Supplementary Table 5. The results of MR-PRESSO after removing outliers.**

| **Metabolite ID** | **Metabolites** | ***p* value in the global heterogeneity test** |
| --- | --- | --- |
| M00063 | cholesterol | 0.925 |
| M01358 | stearate (18:0) | 0.993 |
| M01508 | pantothenate | 0.477 |
| M01564 | citrate | 0.952 |
| M16822 | X-04499--3,4-dihydroxybutyrate | 0.777 |
| M19415 | X-06351 | 0.993 |
| M27728 | glycerol 2-phosphate | 0.775 |
| M32418 | myristoleate (14:1n5) | 0.517 |
| M32425 | dehydroisoandrosterone sulfate (DHEA-S) | 0.816 |
| M32754 | X-11437 | 0.386 |
| M33132 | X-11787 | 0.827 |
| M33610 | X-12189 | 0.935 |
| M33969 | stearidonate (18:4n3) | 0.505 |
| M34245 | X-12645 | 0.906 |
| M37097 | tryptophan betaine | 0.944 |

|  | | **Supplementary Table 6. Estimation of the Steiger direction test from 15 blood metabolites to SSNHL.** | | | | |
| --- | --- | --- | --- | --- | --- | --- |
| **Metabolite ID** | **Metabolites** | | **SNP_r2. exposure** | **SNP_r2. outcome** | **Direction** | **Steiger *p*-value** |
| M00063 | cholesterol | | 1.0032E-03 | 6.15E-10 | TRUE | 9.4991E-03 |
| M01358 | stearate (18:0) | | 9.2512E-04 | 2.97E-10 | TRUE | 1.2607E-02 |
| M01508 | pantothenate | | 7.0242E-04 | 7.31E-10 | TRUE | 3.4028E-02 |
| M01564 | citrate | | 6.1704E-04 | 6.28E-10 | TRUE | 3.7144E-02 |
| M16822 | X-04499--3,4-dihydroxybutyrate | | 2.7475E-04 | 2.98E-10 | TRUE | 1.8682E-01 |
| M19415 | X-06351 | | 8.0945E-04 | 5.94E-11 | TRUE | 7.8834E-02 |
| M27728 | glycerol 2-phosphate | | 1.3713E-03 | 6.46E-10 | TRUE | 8.4856E-03 |
| M32418 | myristoleate (14:1n5) | | 7.3233E-04 | 3.26E-10 | TRUE | 3.9916E-02 |
| M32425 | dehydroisoandrosterone sulfate (DHEA-S) | | 2.7286E-04 | 2.30E-10 | TRUE | 1.6142E-01 |
| M32754 | X-11437 | | 1.0353E-03 | 4.07E-10 | TRUE | 2.3333E-02 |
| M33132 | X-11787 | | 6.9861E-03 | 4.29E-10 | TRUE | 3.7800E-12 |
| M33610 | X-12189 | | 6.2420E-01 | 5.18E-10 | TRUE | 1.1260E-60 |
| M33969 | stearidonate (18:4n3) | | 4.0204E-04 | 2.60E-10 | TRUE | 1.1442E-01 |
| M34245 | X-12645 | | 1.9636E-03 | 2.10E-10 | TRUE | 3.3614E-03 |
| M37097 | tryptophan betaine | | 1.0817E-03 | 9.94E-11 | TRUE | 1.6751E-02 |
